# Live cell analyses of synaptonemal complex dynamics and chromosome movements in cultured mouse testis tubules and embryonic ovaries

**DOI:** 10.1007/s00412-018-0668-7

**Published:** 2018-03-26

**Authors:** Andrea Enguita-Marruedo, Wiggert A. Van Cappellen, Jos W. Hoogerbrugge, Fabrizia Carofiglio, Evelyne Wassenaar, Johan A. Slotman, Adriaan Houtsmuller, Willy M. Baarends

**Affiliations:** 1 Department of Developmental Biology, Erasmus MC University Medical Centre, Rotterdam, The Netherlands; 2 Department of Pathology, Erasmus Optical Imaging Centre, Erasmus MC University Medical Centre, Rotterdam, The Netherlands

**Keywords:** Meiotic prophase, SYCP3, in vitro culture, Spermatocyte, Oocyte, Synaptonemal complex

## Abstract

During mammalian meiotic prophase, homologous chromosomes connect through the formation of the synaptonemal complex (SC). SYCP3 is a component of the lateral elements of the SC. We have generated transgenic mice expressing N- or C-terminal fluorescent-tagged SYCP3 (mCherry-SYCP3 (CSYCP) and SYCP3-mCherry (SYCPC)) to study SC dynamics and chromosome movements in vivo. Neither transgene rescued meiotic aberrations in *Sycp3* knockouts, but CSYCP could form short axial element-like structures in the absence of endogenous SYCP3. On the wild-type background, both fusion proteins localized to the axes of the SC together with endogenous SYCP3, albeit with delayed initiation (from pachytene) in spermatocytes. Around 40% of CSYCP and SYCPC that accumulated on the SC was rapidly exchanging with other tagged proteins, as analyzed by fluorescent recovery after photobleaching (FRAP) assay. We used the CSYCP transgenic mice for further live cell analyses and observed synchronized bouquet configurations in living cysts of two or three zygotene oocyte nuclei expressing CSYCP, which presented cycles of telomere clustering and dissolution. Rapid chromosome movements were observed in both zygotene oocytes and pachytene spermatocytes, but rotational movements of the nucleus were more clear in oocytes. In diplotene spermatocytes, desynapsis was found to proceed in a discontinuous manner, whereby even brief chromosome re-association events were observed. Thus, this live imaging approach can be used to follow changes in the dynamic behavior of the nucleus and chromatin, in normal mice and different infertile mouse models.

**Electronic supplementary material:**

The online version of this article (10.1007/s00412-018-0668-7) contains supplementary material, which is available to authorized users.

## Introduction

In all sexually reproducing diploid species, homologous chromosomes must separate faithfully during the first meiotic division, to generate two haploid cells. Leading up to this event, homologs must form pairs. This requires dynamic movements of chromosomes inside the nucleus, as has been visualized in living rodent spermatocytes using transillumination microscopy already several decades ago (Parvinen and Soderstrom 1976; Salonen et al. 1982). One of the most conspicuous features of meiotic prophase cells that has been observed in almost all analyzed sexually reproducing species to date is the so-called bouquet stage, when telomeres cluster together in the nuclear periphery, and the thread-like chromosomes form a structure that is reminiscent of a bouquet of flowers (Scherthan 2007; Stewart and Burke 2014). In addition, rapid telomere movements occur in both yeast and mouse, and both processes require linkage of the telomeres to the cytoskeleton, through the nuclear membrane (Lee et al. 2012; Lee et al. 2015). These dynamic processes occur hand-in-hand with the initiation of homologous chromosome pairing. Correct chromosome pairing also requires the formation and repair of DNA double-strand breaks, mediated by the SPO11/TOPOVIBL complex (Baudat et al. 2000; Robert et al. 2016; Romanienko and Camerini-Otero 2000), together with additional meiosis-specific proteins. In addition, a DSB-independent role of SPO11 in early homologous chromosome interactions has been described (Boateng et al. 2013). Pairing results in synapsis, defined as the formation of a physical proteinaceous connection between the chromosomes, in a zipper-like fashion along the chromosomal arms. This connecting protein structure is called the synaptonemal complex (SC) and is composed of two lateral elements (LEs, one per homolog) that associate with each other through the transverse filaments (TFs). They overlap in the central region, forming the central element (CE) (Page and Hawley 2004). In mammals, the LEs and their precursors, the axial elements (AEs), are mainly composed of the proteins SYCP2 and SYCP3, while the TFs mainly consist of SYCP1 (Costa et al. 2005; Heyting 1996). The CE contains SYCP1 as well as other proteins like SYCE1, SYCE2, SYCE3, and TEX1 (Costa et al. 2005; Hamer et al. 2006; Schramm et al. 2011).

With the advent of technologies to express fluorescent-tagged proteins, and the means to analyze their expression in living cells using confocal fluorescent microscopy, more possibilities to investigate chromosome movement in detail have arisen. In mice carrying a SYCP3-EYFP transgene driven by a *Pgk2*-promoter, labeled SCs were analyzed in cultured pachytene spermatocytes embedded in a fibrinogen clot for periods of up to 6 h (Morelli et al. 2008). In these nuclei, only subtle movements were detected. More recently (Shibuya et al. 2014) used in vivo DNA electroporation to express fluorescent-tagged SYCP3 and TRF1 in mouse spermatocytes. Cultured cells were analyzed for periods no longer than 10 min. The analysis of the SYCP3 behavior showed rapid movements of chromosomes within the nuclei throughout meiotic prophase. A recent study (Rog and Dernburg 2015) developed in the model organism *C. elegans*, involved analyses of the behavior of the SC in the worms in vivo.

We aimed to study the dynamics of chromosomes and the SC in living mouse spermatocytes or oocytes, in their natural environment (inside the seminiferous tubule or the ovary). We have generated transgenic mice expressing N- or C-terminal fluorescent-tagged SYCP3-mCherry, and developed a method that allows us to culture seminiferous tubules or ovaries during short-term (minutes) and long-term overnight cell-imaging experiments. SYCP3 is an important functional component of the axial/lateral elements of the SC. Disruption of *Sycp3* leads to aberrant chromosome pairing and synapsis, and spermatocytes do not progress further than a zygotene-like stage (Hamer et al. 2008; Royo et al. 2010). Female *Sycp3*^*−/−*^ knockouts show milder defects, and they are subfertile, showing a reduction in litter size (Yuan et al. 2002).

Here, we show that both N-terminal and C-terminal tagging of SYCP3 precludes formation of functional SYCP3 filaments in the absence of endogenous, untagged SYCP3. However, when untagged SYCP3 is also expressed, the tagged proteins can accumulate on the axial elements, exchange dynamically, and do not interfere with normal progression of oogenesis and spermatogenesis. This allowed us to perform detailed analyses of chromosome and nuclear movements during early and late meiotic prophase in oocytes and spermatocytes, respectively. Thereby, this study provides novel insight in nuclear rotation speed, dynamics of bouquet formation, and progression of desynapsis in living mouse meiocytes in a tissue context.

## Results

### Transgenic expression of fluorescent-tagged SYCP3

To drive expression of the transgenes encoding SYCP3 tagged with mCherry at either the N-or C-terminal end of the protein, we used a promoter fragment of *Smc1b*, previously reported to drive specific expression in spermatocytes from leptotene onwards (Fig. S1a) (Adelfalk et al. 2009). We confirmed expression of the tagged protein first on Western blot, using total testis protein extracts from mice of different age. A single band was observed for N-terminally tagged SYCP3 (CSYCP), but C-terminal-tagged SYCP3 (SYCPC) was expressed in two forms, most likely as a result of the presence of two possible start codons in the first exon, as previously reported (Alsheimer et al. 2010). The levels of the two tagged proteins were lower compared to the level of endogenous SYCP3, and expression initiated later during postnatal development (Fig. S1b). Thus, although endogenous SMC1B and SYCP3 are known to display very similar expression patterns in spermatocytes (Dobson et al. 1994; Revenkova et al. 2001), the *Smc1b* promoter fragment used here resulted in reduced and delayed expression of fluorescent-tagged SYCP3 compared to endogenous SYCP3.

### SYCPC and CSYCP locate on the axis of the fully formed SC in spermatocytes, and also on axial elements in oocytes on a wild-type background

Next, we analyzed the localization of both fusion proteins in nuclear spread preparations of spermatocytes and oocytes, whereby the transgene was expressed either on a wild-type or *Sycp3*^*+/−*^ background*.* We detected both endogenous and transgenic SYCP3, making use of anti-SYCP3 antibody, and could selectively analyze the tagged protein by detecting the fluorescent signal from the mCherry moiety.

**Fig. 1:**
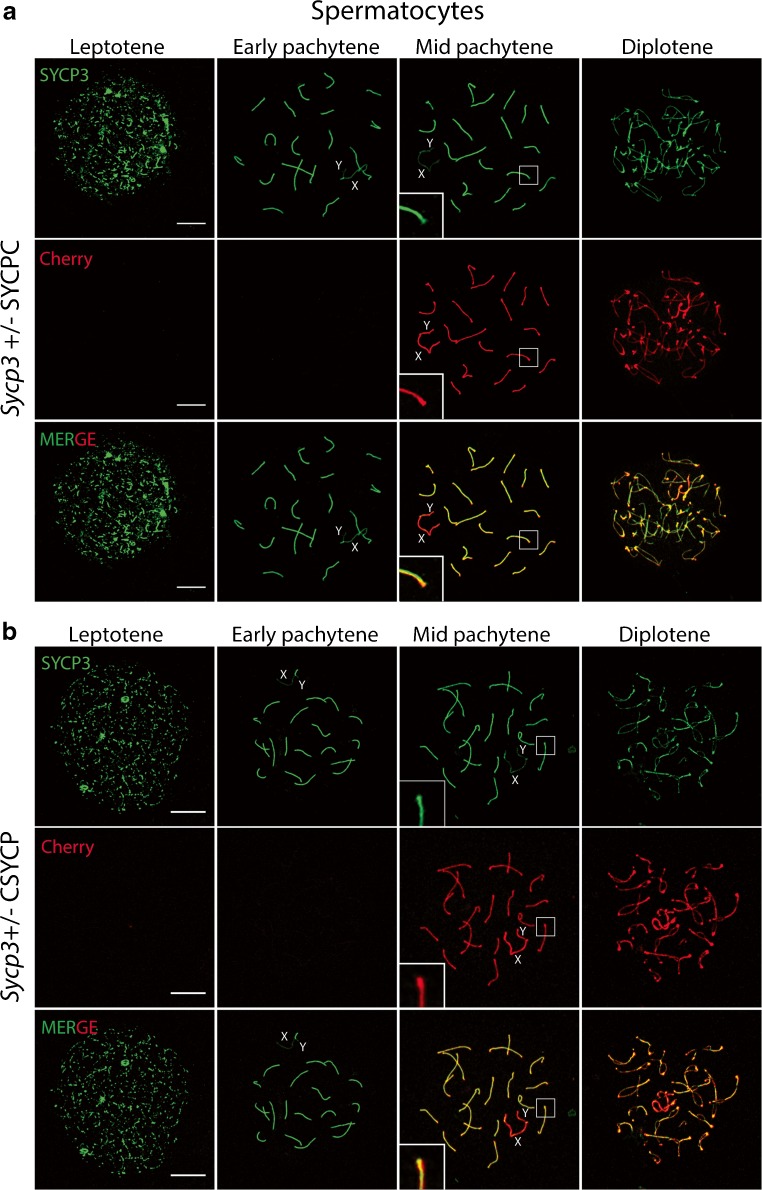
Expression of SYCPC and CSYCP in wild-type spermatocytes. Immunostaining of SYCP3 (green) on *Sycp3* ^*+/−*^ SYCPC (**a**) and *Sycp3*^*+/−*^ CSYCP (**b**) spermatocyte nuclei at different stages of meiotic prophase. The red signal represents the mCherry signal (no antibody detection). Scale bar 10 μm

In males, SYCPC as well as CSYPC were detected from pachytene onwards, along the lateral elements of the SC, colocalizing with endogenous SYCP3 (Fig. 1). Interestingly, both fusion proteins displayed a signal of higher intensity at telomeric ends of the SC of the autosomes (Fig. 1a, b, enlargements in mid pachytene) and also along the axial elements of the X and Y chromosomes (Fig. 1a, b, mCherry signal in mid pachytene). Enrichment of fusion protein on the XY pair was most evident for CSYCP (Fig. 1b, mCherry signal in mid pachytene). In contrast, the overall SYCP3 signal (Fig. 1a, b, SYCP3 in mid pachytene, consisting of endogenous as well as tagged protein) was lower along the non-synapsed XY axes, compared to the synapsed autosomes, and no thickening was observed at the SC ends.

**Fig. 2:**
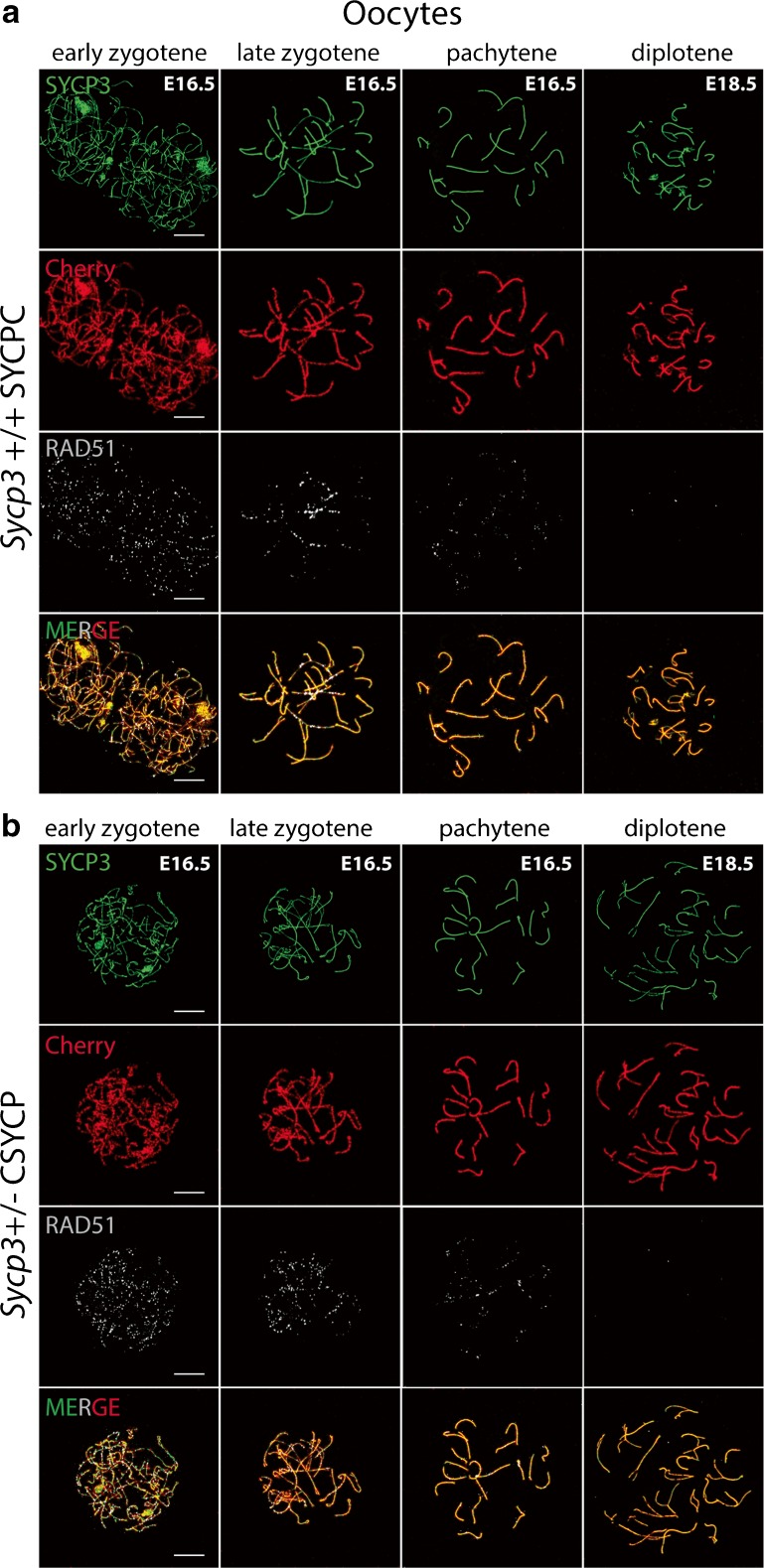
Expression of SYCPC and CSYCP in wild-type oocytes. Immunostaining of SYCP3 (green) and RAD51 (white, pseudo-color from infrared) on *Sycp3* ^+/+^ SYCPC (**a**) and *Sycp3* ^*+/−*^ CSYCP (**b**) oocyte nuclei from embryonic ovaries isolated at E16.5 (early and late zygotene and pachytene) and E18.5 (diplotene). mCherry (red) is visualized directly (no antibody detection). Scale bar 10 μm

In embryonic ovaries, CSYCP and SYCPC were detected in oocyte nuclei from leptotene (embryonic day 16.5 (E16.5) until diplotene (E18.5) stages (Fig. 2a, b, staging of oocytes is described in the “Materials and methods” section). Also here, the tagged proteins colocalized with endogenous SYCP3. We did not detect either fusion protein in ovaries isolated from embryos younger than E16.5. No differences were observed between the patterns of the two fusion proteins, and also not between the overall SYCP3 signal and that of the mCherry-tagged protein only. The fact that both SYCPC and CSYCP localize to axial elements in leptotene and zygotene oocytes, but not in such early spermatocyte nuclei, is most likely due to the fact that the *Smc1b* promoter fragment used in our transgene constructs is not yet active in leptotene and zygotene spermatocytes in our models (see the “Discussion” section).

### mCherry-tagged SYCP3 does not interfere with normal meiotic progression but cannot rescue the *Sycp3* knockout phenotype

**Fig. 3:**
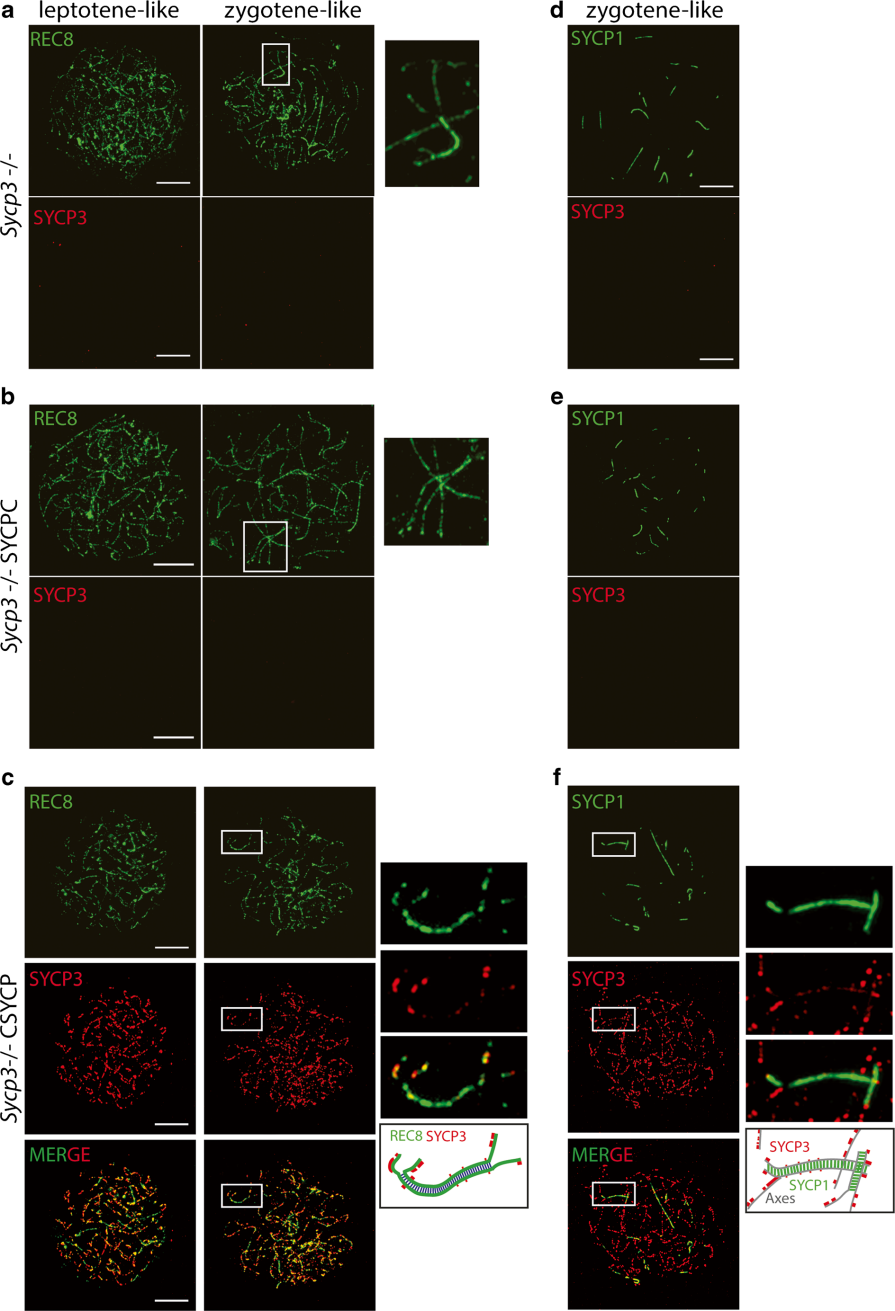
Expression of SYCPC and CSYCP in Sycp3^−/−^ spermatocytes. Immunostaining of REC8 (green) and SYCP3 (red) (**a**–**c**) or SYCP1 (green) and SYCP3 (red) (**d**–**f**) on *Sycp3* ^−/−^ (**a**, **d**), *Sycp3*^−/−^ SYCPC (**b**, **e**), and *Sycp3*^*−/−*^ CSYCP (**c**, **f**) spermatocyte spreads. Enlarged regions (indicated by white boxes) are shown on the right (**a**–**c**, **f**). Below the enlargements in **c** and **f**, a schematic drawing clarifies the merged image. Scale bar 10 μm

Tagging a protein in vivo may interfere with its normal function. In addition, regulation of expression of our transgene differs from that of endogenous SYCP3, which may also influence its degree of functionality. To assess whether the expression of the two SYCP3 fusion proteins interfered with normal progression of meiosis, we assessed overall fertility (Fig. S2a), crossover frequency (Fig. S2b), and persistence of DSBs in late meiotic prophase (Fig. S2c) in males carrying the transgene in addition to one or two wild-type *Sycp3* alleles. None of these parameters were affected. To investigate to what extent the SYCPC and CSYCP proteins were capable of functionally replacing endogenous SYCP3, we analyzed the meiotic phenotype of *Sycp3*^*−/−*^ male and female mice, in the presence or absence of SYCPC and CSYCP. Prophase substages were determined using the known stage-specific localization pattern of the meiosis-specific cohesin component REC8, which mostly colocalizes with SYCP3 in wild-type spermatocytes and oocytes (Lee et al. 2003). In male *Sycp3*^*−/−*^, *Sycp3*^*−/−*^ SYCPC, and *Sycp3*^*−/−*^ CSYCP spreads, we observed leptotene and zygotene-like stages (Fig. 3a–c), but no cells in which complete synapsis was achieved. In addition, no SYCPC protein was detected in *Sycp3*^*−/−*^ SYCPC males, upon staining with anti-SYCP3 (Fig. 3b). In contrast, CSYCP could be detected as small patches along the axial elements (Fig. 3c) in 67% of the nuclei analyzed (*n* = 52 nuclei, 2 mice). When CSYCP was present, it mostly colocalized with REC8 (Fig. 3c, enlargements). Immunodetection of SYCP1 (marker of synapsis) revealed that the degree of synapsis in *Sycp3*^*−/−*^ CSYCP, *Sycp3*^*−/−*^, and *Sycp3*^*−/−*^ SYCPC spermatocytes was similar (Fig. 3d–f). Interestingly, CSYCP accumulation was less apparent on (heterologously) synapsed SC fragments compared to unsynapsed axial elements (Fig. 3f, enlargements). To further evaluate functionality of SYCPC and CSYCP, we analyzed if our tagged SYCP3 proteins would alter the pattern of SYCP2 accumulation on the knockout background, since it has been reported that SYCP2 fails to localize to the axes upon knockout of *Sycp3* (Pelttari et al. 2001), and vice versa (Yang et al. 2006). However, in contrast to the published results, we observed that SYCP2 was still present on the axes of *Sycp3*^*−/−*^ spermatocytes, and also of *Sycp3*^*−/−*^ SYCPC and *Sycp3*^*−/−*^ *CSYCP* spermatocytes (Fig. S3a,b). Identical results were obtained with two different antibodies targeting SYCP2. Thus, it appears that SYCP2 localization does not depend on SYCP3 expression and is not influenced by the presence of mCherry-tagged SYCP3. Together, the data indicate that the *Sycp3*^*−/−*^ phenotype is not rescued by either transgene in males.

**Fig. 4:**
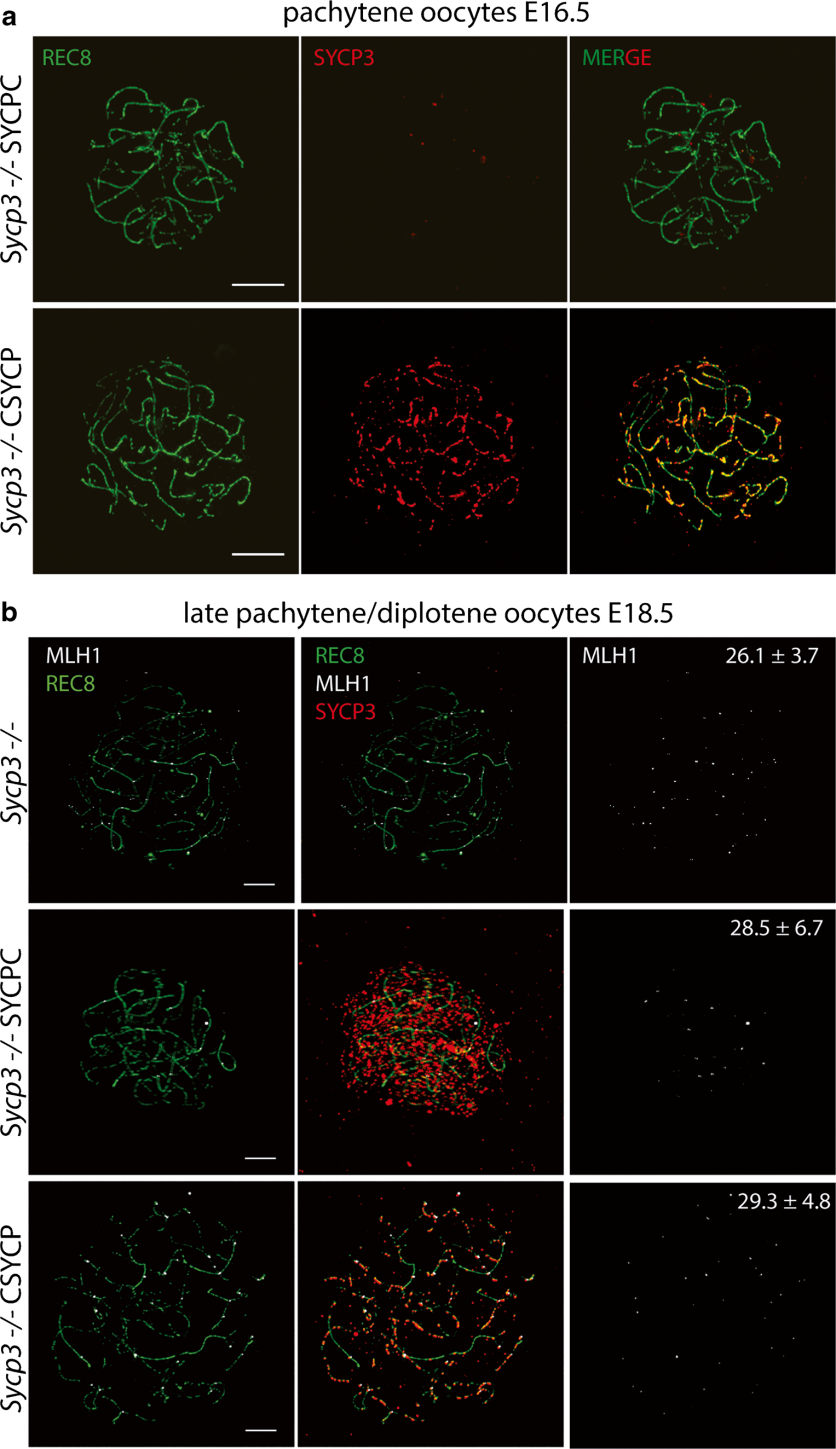
Expression of SYCPC and CSYCP in *Sycp3*^−/−^ oocytes. **a** Immunostaining of REC8 (green) and SYCP3 (red) on *Sycp3*^−/−^ SYCPC and *Sycp3*^−/−^ CSYCP pachytene oocyte nuclei at E16.5. **b** Immunostaining of REC8 (green), SYCP3 (red), and MLH1 (white, pseudo-color from infrared) on *Sycp3*^−/−^, *Sycp3*^−/−^ SYCPC, and *Sycp3*^*−/−*^ CSYCP oocyte nuclei at E18.5. Mean of MLH1 foci ± SD are displayed in the images, *n* = 7 nuclei for *Sycp3*^*−/−*^ and *Sycp3*^*−/−*^CSYCP, *n* = 6 nuclei for *Sycp3*^*−/−*^SYCPC

We then determined if SYCPC and CSYCP proteins could functionally replace SYCP3 in females. Similar to what was observed in males, SYCPC did not localize to axial elements in the absence of endogenous SYCP3 in E16.5 leptotene and zygotene oocytes (Fig. S4a) but showed focal accumulation on chromatin (not in association with axial elements) at later stages, in late pachytene/diplotene-like oocytes at E18.5 (Fig. 4b). CSYCP already accumulated on the chromatin at leptotene and zygotene in E16.5 *Sycp3*^*−/−*^ CSYCP oocytes (Fig. S4b) and clearly displayed axial localization when cells reached a pachytene-like stage (E16.5, Fig. 4a and S4b; E18.5, Fig. 4b). However, the protein covered only part of the axes visualized by anti-REC8. During normal meiotic prophase progression, RAD51 foci numbers (indicative of DSB repair sites) gradually decrease and disappear in diplotene. In contrast, RAD51 foci are retained in *Sycp3*^*−/−*^ pachytene and diplotene oocytes (Wang and Hoog 2006). The presence of SYCPC or CSYCP did not reduce the number of persisting RAD51 foci in *Sycp3*^*−/−*^ pachytene and diplotene oocytes at E18.5 (Fig. S5a-c). In addition, MLH1 foci numbers (indicative of crossover sites (Kolas and Cohen 2004; Moens et al. 2002)) did not differ between the genotypes (Fig. 4b). Thus, the tagged proteins do not rescue the *Sycp3*^*−/−*^ oocyte phenotype either.

### Identification of prophase substages in living spermatocytes and oocytes expressing CSYCP

**Fig. 5:**
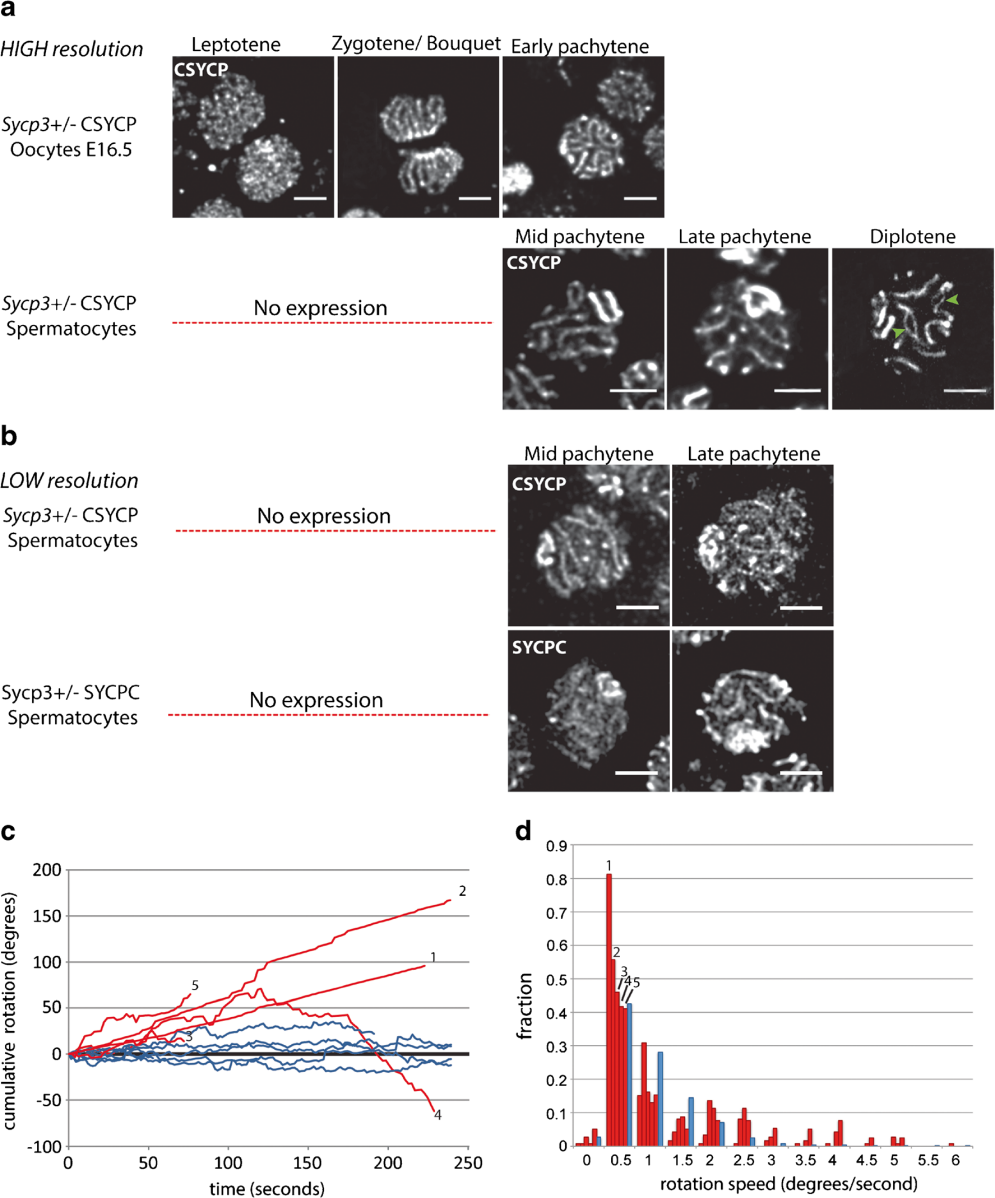
Live imaging: CSYCP in oocytes and spermatocytes (**a**) and CSYCP and SYCPC in spermatocytes (**b**). High-resolution images are depicted in **a** (obtained using the SP5 confocal microscope, except for the diplotene spermatocyte (lower panel), which was generated using the Airy-scan detector). Green arrowheads in the diplotene spermatocyte indicate desynapsed regions. Low-resolution images are shown in **b** (SP5 confocal microscope). The low-resolution images display more background and less clear axes. No expression indicates absence of fusion protein expression during leptotene and zygotene stages in spermatocytes. Scale bar 5 μm. **c** Cumulative rotations of oocytes (red) and spermatocytes (blue) plotted against time (seconds). Clockwise rotations are added as positive values (Y axis), while counterclockwise rotations are negative values. **d** Relative frequencies of rotation speeds of each oocyte (red) and all analyzed spermatocytes (all speeds included, distributions of individual spermatocytes were similar, blue). Same oocyte in **c** and **d** is indicated with the same number. *N* = 5 oocytes and 6 spermatocytes

We setup a method to allow time-lapse analyses of oocytes and spermatocytes in cultured embryonic ovaries and tubule fragments, respectively. We used females (E16.5) and males carrying the CSYCP transgene on the wild-type background. The organs or tubule fragments were cultured in a gelatinous protein mixture (basement membrane extract, BME) to maintain three-dimensional structures (see the “Material and methods” section for more details). Stages from leptotene to pachytene were observed in oocytes (Fig. 5a, upper panel) and identified as follows: leptotene nuclei displayed thin axes, and their telomeres were dispersed in the nucleus (Fig. 5a, upper panel, leptotene). Clustering of telomeres in the periphery of the nucleus was observed during the bouquet stage (Fig. 5a, upper panel, zygotene/bouquet). Subsequently, the thickness of the axes varied upon synapsis progression. In pachytene oocytes, all the axes were synapsed (Fig. 5a, upper panel, early pachytene). In the same way, completely synapsed axes were observed in pachytene spermatocytes in *Sycp3*^*+/−*^ CSYCP males (Fig. 5a, lower panel). In late pachytene, thicker chromosome ends and brighter XY axes were observed (Fig. 5a, lower panel, late pachytene). Finally, partially desynapsed axes were observed in diplotene spermatocytes (Fig. 5a, lower panel, diplotene). Although we mainly focused on CSYCP for our time-lapse experiments, similar in vivo observations were made in cultured ovaries and testis tubules from mice expressing SYCPC (see for example Fig. 5b, SYCPC and CSYCP patterns in spermatocytes (imaged at somewhat lower resolution compared to Fig. 5a)).

### Synaptonemal complex movements are rapid during prophase

Movements were highly dynamic during the entire meiotic prophase, in both male and female meiocytes. Two types of movements were observed: rotation of the nucleus itself and movement of the SC inside the nucleus. The rapid movements prompted us to perform an experiment whereby we recorded a single plane with a time lapse of only 1.5–2 s (this short time frame in between recordings precluded imaging multiple planes). To estimate nuclear rotation speed, we then selected single zygotene oocyte and pachytene spermatocyte nuclei that could be traced in time and calculated rotation angles between two consecutive time points as described in the “Materials and methods” section. We then plotted the cumulative rotation angle against time, whereby rotations in clockwise direction were considered as positive angles and counterclockwise rotations as negative angles (Fig. 5c). Interestingly, oocytes rotated for long time periods in a single direction, whereas spermatocytes presented a kind of “wiggling” movement. However, the oocytes displayed more variability in rotational speed frequency distribution (Fig. 5d), in contrast to the spermatocytes, for which the majority of measured rotation speeds were relatively small (71% of speeds between 0.5 and 1.5 degrees/s).

The extensive rotation of the nucleus itself, together with the SC movements, increased complexity to such an extent that we could not follow movement of individual autosomal SCs in oocytes and spermatocytes. The XY axis in pachytene and diplotene spermatocytes was more easily traceable because of its high mCherry signal. Taking the center of the XY axis as reference, we found that it moved with a speed of 24 ± 15 nm/s (*n* = 10 nuclei). This displacement is the combined result of nuclear rotation and independent movement of the XY within the nucleus.

### Meiotic prophase progression, including bouquet formation, is synchronized between oocytes in the same cyst

**Fig. 6:**
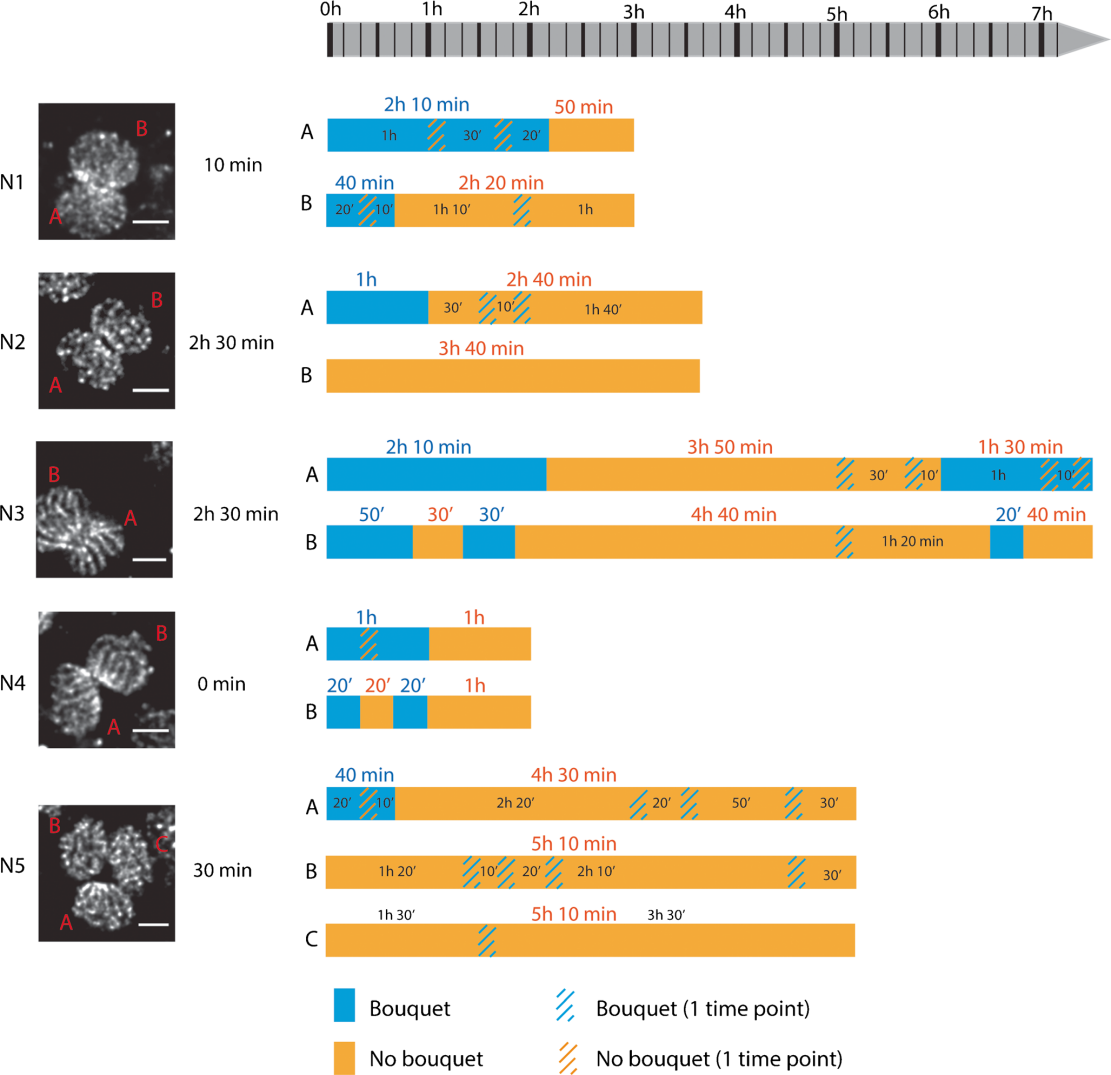
Bouquet progression in oocytes. Five groups of zygotene nuclei of two different experiments (experiment 1: N1 and N4; experiment 2: N2, N3 and N5) were analyzed. Nuclei are ordered from early to late zygotene, from top to bottom of the figure. An image at t0 is showed for N1-N4. For N5, t5 was chosen, to be able to show the three nuclei in the same plane. Scale bar 5 μm. The amount of time passed up to the first observed bouquet is indicated before each time bar. Blue bars indicate periods during which a bouquet stage was observed; orange bars indicate periods in which no bouquet was observed for longer than 10 min. Orange diagonal lines indicate loss of clear bouquet configuration at only a single time point; blue diagonal bars indicate apparent bouquet organization of chromosomes at only a single time point

The fast movements of chromatin and nuclei in early prophase oocytes precluded detailed analyses of the progression of synapsis of individual chromosomes in time. Nevertheless, we could study some other general features in these oocytes. Frequently, we observed two, or sometimes even three, oocytes that moved together as a single unit during the whole recording (from several minutes until a maximum of 10 h in the overnight experiments). The two or three connected oocytes were always found in the same meiotic stage, indicating synchronized progression through meiosis (videos 1, 2 and 3). The telomeres could be distinguished because of their higher fluorescent signal compared to the rest of the axes, allowing us to observe events of clustering/dissolution of telomeres during the zygotene stage. Clustering and dissolution events sometimes followed each other in rapid succession, even within a few seconds of each other (video 4, bouquet formation can be clearly observed at *t* = 6 s in the upper nucleus. Dissolution occurs immediately thereafter). These brief clustering events could be unstable or random events, and we expected that a real functional bouquet event would be maintained for a longer time period. For this reason, we also analyzed bouquet progression in our overnight experiments. In order to prevent problems of bleaching during the long experiment, but to be able to follow nuclei despite the high movement frequency, an interval of 10 min was chosen for these time-lapse 3D recordings. The fast movements of the chromosomes and nuclei, together with the low resolution in Z, prevented us from obtaining a high-resolution 3D image of the stacks. Still, we could identify bouquet stages using two criteria: clear linear organization of several telomeres next to each other in one of the planes of the z-stack and the continuation of this situation during at least two consecutive time points. This second criterion was also applied to the non-bouquet situation. The observations on different groups of nuclei (N) (N1 and N4, experiment 1; N2, N3 and N5, experiment 2) are summarized in Fig. 6. The nuclei were ordered according to their degree of meiotic progression, whereby N1 and N2 were at the earliest stages of prophase, followed by N3, N4, and N5, which represented more and more advanced stages of zygotene development. The duration of bouquet configuration varied among these nuclei, ranging from 20 min (by definition the shortest duration possible) to 2 h and 10 min. The behavior of the nuclei within one cyst also varied. In N2 and N5, we observed bouquet formation in only one of the two nuclei. In N1, one bouquet is dissolved earlier than the other, but in N3 and N4, the nuclei in the cyst behaved synchronously and the bouquet configuration appeared and disappeared approximately at the same time. Some cysts (nucleus B of N3, and nucleus B of N4) exhibited two cycles of telomere clustering, dissolution, and reclustering. Single time point observations of dissolution or clustering could also frequently be observed. As an example, the complete sequence of images for N3 is shown in Fig. S6. Interestingly, when bouquets of two nuclei were present in the same syncytium, they always appeared as if facing each other.

### Axis separation starts in interstitial regions and progresses towards the telomeres of the chromosomes

**Fig. 7:**
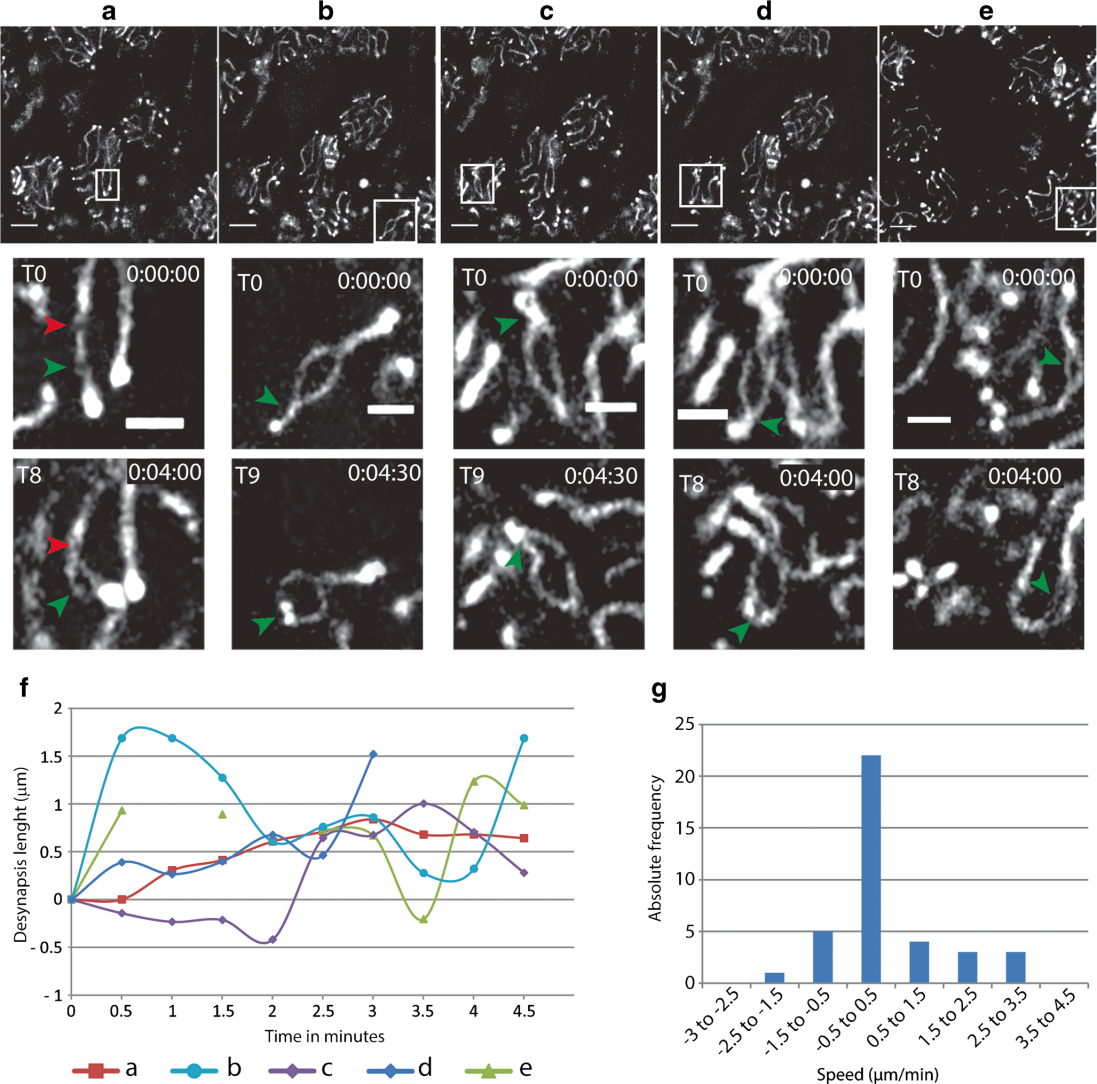
Analyses of axis separation in diplotene spermatocytes. **a**–**e** Desynapsis progression in five different bivalents of *Sycp3* ^*+/−*^ CSYCP diplotene spermatocytes. CSYCP is visualized in gray. An overview of the whole field of view at T0 is shown in the upper panels (scale bar 5 μm). Below each image, enlargements of the bivalent that desynapses (indicated by a boxed area) are shown at T0 and at T8 or T9 (4 or 4. 5 min later). Scale bar 2 μm. **f** Progression of desynapsis in time for each bivalent. Note that bivalent e (light green) could not be measured at *t* = 1 min and *t* = 2 min, because it moved outside the field of view (see video 9). **g** Frequency distribution of the desynapsis (positive)/resynapsis (negative) speeds of the five bivalents. Speeds were measured as described in the “Materials and methods” section. All speeds of all bivalents were analyzed together

In diplotene spermatocytes, it was possible to follow the process of axis separation (further referred to as desynapsis) for single chromosome pairs in five bivalents. Desynapsis initiated in the interstitial region of the chromosomes and progressed towards the telomeres (Fig. 7a–e, videos 5-9, representing bivalent a–e, respectively). In the bivalent shown in Fig. 7a and video 5, desynapsis progressed from the desynapsis initiation point indicated with a green arrowhead, while there was no progression of desynapsis from the area indicated with the red arrowhead, indicating that desynapsis does not proceed in a similar fashion from each initiation point on a single bivalent. In all nuclei, desynapsis was discontinuous and even resynapsis was observed for two bivalents (Fig. 7f, nucleus b and e, videos 6 and 9). This can also be inferred from the desynapsis length plotted against time for each individual bivalent that was traced (Fig. 7f). In addition, the frequency distribution of the measured speeds visualizes the variability of the velocity (Fig. 7g: desynapsis (positive values) and resynapsis (negative values)). Around 60% of the measured velocities were included in the interval between − 0.5 and 0.5 μm/min, indicating that most of the time period during which the measurements took place was taken up by only minute changes in the degree of axis separation. When visible desynapsis/resynapsis took place, the most frequently measured speed was around 1 μm/min for both situations (11 and 13%, respectively). Faster velocities were rare during resynapsis, but relatively frequent during desynapsis (16% of the values were between 1.5 and 3.5 μm/min). In addition, desynapsis events occurred more frequently (27% of the measured speeds) than resynapsis (16%), explaining the overall increase in desynapsis over time for all analyzed bivalents.

### CSYCP and SYCPC can be recruited to the lateral elements of the SC

**Fig. 8:**
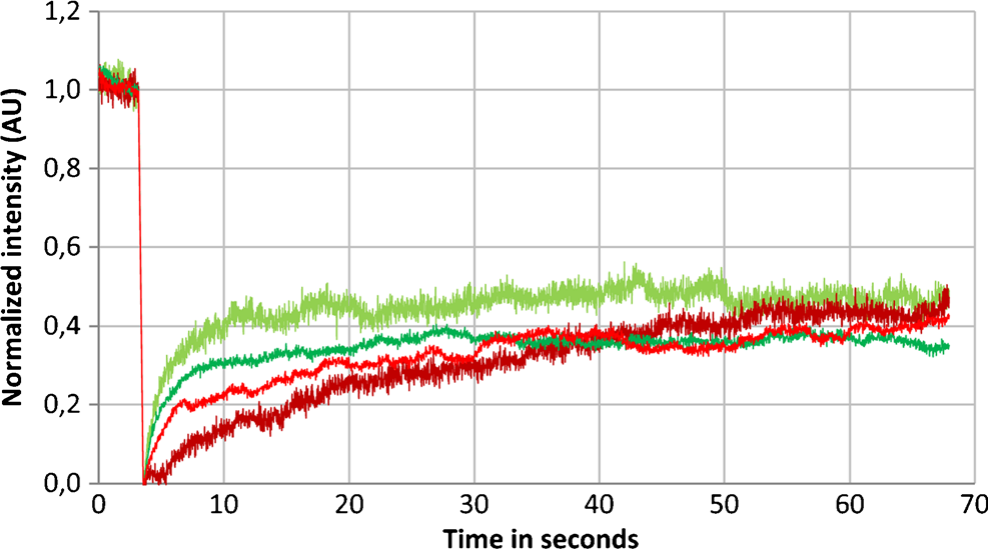
Fluorescence recovery after photobleaching of SYCPC and CSYCP in autosomes of pachytene spermatocytes. Each line represents the averaged and normalized fluorescent recovery of at least ten spermatocyte nuclei of one mouse testis. The results obtained of two *Sycp3* ^*+/−*^ SYCPC (green) and two *Sycp3* ^*+/−*^ CSYCP (red) males are plotted against time. Total time recording after bleaching 1 min. Time lapse 0.020 s

To determine if CSYCP and SYCPC were stably bound to the lateral elements in spermatocytes, fluorescence recovery after photobleaching (FRAP) was performed. The recovery of at least 10 individual spermatocytes was analyzed per transgenic mouse, expressing either SYCPC or CSYCP in the presence of untagged protein. A single strip that spanned the middle part of the nucleus was photobleached. To reduce technical artifacts due to the high frequency of nucleus and chromosome movement, the nucleus was imaged with a small interval of 0.020 s, and a total time of 1 min, and the XY was never included inside the bleached strip. The average and normalized fluorescence recovery of CSYCP and SYCPC was plotted against time (Fig. 8). A final recovery of 40% was observed within 1 min for both CSYCP and SYCPC in the two experiments.

## Discussion

### Both N- and C-terminal fusion of mCherry to SYCP3 affects filament formation

Neither transgene could rescue the aberrant chromosome pairing and frequent asynapsis that is observed upon knockout of *Sycp3* in the male mice (Yuan et al. 2000). However, while no SYCPC was detected on the axes of spermatocytes and oocytes on a *Sycp3* knockout background, CSYCP could form small patches. Recent results from in vitro analyses have indicated that SYCP3 may assemble in tetramers (Syrjanen et al. 2014). Self-interactions among these tetramers (self-assembly), depending on the last six amino acids of the C-terminal of SYCP3, mediate SYCP3 filament formation in vitro. In these in vitro experiments, the N-terminus appeared to be more important for DNA binding (Syrjanen et al. 2014). We assume that tagging the C-terminus of SYCP3 (SYCPC) precludes de novo assembly of the protein on chromosomal axes but still allows it to associate with DNA at random sites in the chromatin, in the absence of endogenous SYCP3. On the other hand, the intact C-terminus of CSYCP allows it to properly localize to the axes where oligomers can assemble with each other. However, because the N-terminal tag interferes with higher order structures and/or because the DNA-binding capacity is affected, only small filaments can be formed. Although we cannot exclude that the added mCherry tags affect overall protein folding, we think that this is unlikely, given the fact that both proteins are recruited properly to the SC on the wild-type background. The aberrant chromatin localization pattern of both SCYCP and SYCPC in the absence of endogenous SYCP3 already explains their lack of functionality. However, in addition, the aberrant timing of transgene activation during meiotic prophase, as revealed on the wild-type background (see below), may also contribute to the lack of rescue of the *Sycp3* knockout phenotype.

### SYCPC and CSYCP can accumulate on the axes of the SC and their expression does not interfere with fertility

We observed that, on the wild-type background, both CSYCP and SYCPC localize to the axes of the SC, together with endogenous SYCP3, from pachytene onwards in male mice, and from leptotene onwards in embryonic ovaries at E16.5 or later. The lack of SYCPC and CSYCP accumulation on axial elements in wild-type early spermatocytes was unexpected, since the *Smc1beta* promoter fragment that we used has been reported to drive expression from leptotene onwards (Adelfalk et al. 2009). The expression pattern for both transgenes in our mouse models follows the overall pattern of transcriptional activity that has been reported for male meiotic prophase: a general transcriptional inactivation occurs in early stages of male meiosis (leptotene, zygotene and early pachytene), followed be reactivation from mid pachytene onwards (Kierszenbaum and Tres 1974; Monesi 1964; Page et al. 2012). Thus, it is possible that the transgenes are situated in regions that are subject to this global inactivation mechanism, and therefore can only be transcribed from pachytene onwards in males, when global transcription is reactivated. However, we cannot exclude that the observed expression patterns are inherent to the promoter fragment used. In females, expression of the fusion protein was also observed later than expected, and while endogenous SYCP3 can be detected from E13.5 onwards, the tagged proteins were only expressed from E16.5 onwards, when leptotene oocytes still form. Since the tagged proteins are expressed (and incorporated into the LEs) only from pachytene onwards in males, and FRAP analyses showed a final recovery of 40% for both SYCPC and CSYCP during pachytene, we can conclude that tagged SYCP3 can be recruited to already synapsed axes and exchange there. These data might suggest that a mixed filament of endogenous and tagged SYCP3 forms, whereby endogenous SYCP3 protein also exchanges. However, we cannot exclude that the tagged proteins are loosely associated and do not exchange with endogenous SYCP3.

Remarkably, both *Sycp3*^*+/−*^ SYCPC and *Sycp3*^*+/−*^ CSYCP spermatocytes presented a stronger mCherry signal on the (largely) unsynapsed XY compared to the synapsed autosomes. Also, the telomeric regions of the chromosomal axes are enriched for the fusion proteins. Using antibody staining, to detect both endogenous and tagged SYCP3, we observed a dimmer signal on the XY due to the single axes, and no enrichment at the telomeres in mid pachytene, similar to the wild-type pattern. This difference might be explained by increased exchange between endogenous SYCP3 and SYCPC/CSYCP in these regions, if this type of exchange occurs. In this situation, possible differences in the turnover of tagged SYCP3 and endogenous SYCP3 would be relevant. As pachytene progresses, the relative amount of tagged SYCP3 compared to endogenous SYCP3 may increase, allowing an increase in the amount of tagged protein in regions that somehow are exchanging more SYCP3. In the absence of exchange of tagged SYCP3 with endogenous SYCP3, the data can also be explained by the presence of more binding sites for the fusion protein, in the unsynapsed XY and telomeric regions, compared to the rest of the SC, in combination with an overall (much) lower expression of the fusion protein compared to endogenous protein, masking this effect in the immunostainings. The extra accumulation of SYCPC and CSYCP on the axial/lateral elements of the XY pair adds another interesting feature to the structural properties the XY pair, in addition to the described relatively short chromatin loops and long lateral elements of the pseudoautosomal region of the XY pair (Kauppi et al. 2011), which is the part that actually displays stable synapsis.

Wild-type males and females expressing either transgene displayed normal progression through meiotic prophase, and overall fertility was not affected. In addition, we observed no detrimental effects of the long-term and short imaging experiments, since the overall morphology of the nuclei and the SC had not changed, and cellular movements kept occurring within the ovaries and tubule fragments that were cultured for short or long (overnight) periods. In the tubule cultures, contractions of the tubules also still occurred after overnight imaging. Thus, despite the fact that the proteins cannot fully replace endogenous SYCP3, our system provides a tool to study meiosis in living cells, in the natural context of the seminiferous tubule or the embryonic ovary.

### Nuclear rotations and chromosome movements

We have observed two types of movements inside the cultured seminiferous tubules and the ovaries of our transgenic mouse models: the rotation of the nucleus itself and movement of the SC inside the nucleus. The analysis of the movement of the SC inside the nucleus revealed an average speed of the SC of the XY of 24 ± 15 nm/s. Other groups measured telomere velocity in mouse pachytene cells and observed 130 nm/s (Shibuya et al. 2014) or 36 ± 14.8 nm/s (Lee et al. 2015). The latter result nicely fits our data; a somewhat faster movement of the telomeres compared to the rest of the chromosome is to be expected, since the movement of the telomeres along the nuclear envelope may displace also the rest of the chromosome, but to a lesser extent. Related to this, in maize pachytene cells, it has also been observed that chromosome ends travel faster than interstitial regions of the same chromosomes (Sheehan and Pawlowski 2009). Whether the chromosome movements that we observed in late meiotic prophase have any function is unknown. However, it seems to be an evolutionary conserved feature, since it has also been described for budding yeast, where telomere speed was similar in paired and non-paired configurations (Scherthan et al. 2007; Conrad et al. 2008; Koszul et al. 2008). For budding yeast, it has been suggested that chromosome movements after chromosome pairing could be required to help to prevent topological entanglements or to eliminate them if they persist up to the pachytene stage (Koszul et al. 2008). Alternatively, late telomere movements could have a role in late steps of recombination like Holliday junction formation and resolution in pachytene (Scherthan et al. 2007).

Existence of nuclear rotation was also mentioned in previous studies in rat spermatocytes (Parvinen and Soderstrom 1976), but not further characterized. We have analyzed the rotation of the nuclei in zygotene oocytes and pachytene spermatocytes, and we have observed longer time periods of rotation in a single direction in oocytes in early meiotic prophase, compared to pachytene spermatocytes. This observation is consistent with the fact that rotational movements accompany chromosome pairing, and the idea that perhaps concerted movements of telomeres to the same site on the nuclear envelope induce rotations of the entire nucleus (Scherthan et al. 1996). Indeed, the function of the rotational movements in promoting pairing seems to be conserved in evolution. However, the pattern of these movements appears to vary among organisms. In maize meiocytes, oscillations back and forth are the most common type of nuclear rotational movement, although rotations in a single direction (small and large) have also been observed (Sheehan and Pawlowski 2009). In fission yeast, oscillations of the entire nucleus between the two poles (horsetail movements) start just after bouquet formation. The impairment of these movements results in reduced pairing of the homologous chromosomes (Yamamoto and Hiraoka 2001; Saito et al. 2005; Chacon et al. 2016). Interestingly, horsetail movements continue after pairing has been achieved. Their inhibition after pairing produces mis-segregation of the chromosomes during the first meiotic division. Such late oscillations may prevent prolonged associations of the homologous chromosomes once pairing has been achieved, which may lead to irresolvable recombination intermediates and segregation failure (Chacon et al. 2016). The “wiggling” of late spermatocyte nuclei could assist in a similar process, also in combination with the movement of the chromosomes.

### The bouquet stage in mouse oocytes is dynamic

It is known that primordial germ cells form cysts connected by intercellular bridges in the ovaries of E11.5 to E17.5 mice (Pepling 2006; Pepling and Spradling 1998). Accordingly, we have observed that E16.5 oocytes frequently formed groups of two, and occasionally three, nuclei that localized in a single cytoplasm. Bouquet formation within such syncytia was frequently synchronized and the opposite localization of the telomere clusters gave such nuclei a “kissing” appearance. The intercellular bridges could be involved in this synchronization, since bundles of microtubules have been shown to traverse intercellular bridges (Pepling and Spradling 1998). These microtubules are implicated in cellular transport among cells in a cyst (Pepling and Spradling 1998), but they could also play an important role in the synchronization of bouquet formation and connect the cytoplasmic components of the proteins that mediate the clustering of telomeres in association with the nuclear membrane.

In female mice, bouquet formation has been reported to peak at mid to late zygotene and to persist in a substantial proportion of pachytene oocytes (Tankimanova et al. 2004). In accordance with this observation on fixed samples, the living nuclei in which we observed a bouquet were most often in a mid-late zygotene stage. Because of the limitations of our imaging system, we cannot exclude that we underestimate the duration of the bouquet stage. However, what is clear is that the bouquet stage is highly dynamic. During the recordings, some nuclei exhibited two cycles of telomere clustering, dissolution, and reclustering. In some nuclei within a syncytium, the bouquet of one nucleus dissolved while in the other it remained, followed by reclustering of the telomeres in the nucleus that had previously lost its bouquet configuration. In other syncytia, clustering and dissolution cycles occurred more synchronously and sometimes a dissolution/reclustering cycle could be very rapid (around 20 min). Maybe some events of dissolution occur when not all the chromosomes are synapsed or when (partial) non-homologous associations are present that activate a feedback mechanism that triggers reformation of the bouquet, to allow complete synapsis of the remaining unsynapsed axes. Alternatively, or in addition, telomere declustering and reclustering events could be important to solve whole chromosome entanglements, known as “interlocks” that are present during synapsis but absent by the end of pachytene (and therefore resolved) (Zickler and Kleckner 2015; Zickler and Kleckner 2016).

### Desynapsis

Finally, we were also able to follow axis separation, representing desynapsis in male diplotene spermatocytes. In these cases, we observed initiation of desynapsis in the interstitial region of the chromosomes and progression of desynapsis towards the telomeres. Since we could only follow five bivalents, we cannot exclude that desynapsis events might also start from the telomeres. The speed of desynapsis varied; we observed discontinuous progression, and even brief events of resynapsis, indicating that desynapsis occurs in bursts and may also halt for a certain time period. Given the overall normal development of spermatocytes even during overnight imaging experiments, and the observation that multiple desynapsis or resynapsis proceedings could be traced for a single bivalent, we infer that our settings do not interfere with overall chromosome behavior during meiotic prophase. If we estimate the total length of the SC to be around 200 μm (Baarends et al. 2003), it might be expected that desynapsis would not take much longer than 200 min, since multiple bivalents are usually observed to desynapse simultaneously. However, analyses of fixed samples has indicated that in mouse, diplotene lasts around 3 days (Oud et al. 1979). Thus, it can be concluded that the duration of desynapsis is limited by factors other than the actual constraints of the desynapsis process itself.

In summary, we have used the mCherry SYCP3 and SYCP3 mCherry male and female mice to study synaptonemal complex and chromosome dynamics in vivo, for the first time in the natural context of the seminiferous tubule or the ovary, during an imaging time frame of several hours. We have focused on bouquet formation, nuclear rotation, and desynapsis events. In addition to providing new insights in the dynamics of chromosome behavior in wild-type meiocytes, our method can be used as a tool to study alterations in the chromosome dynamics of meiocytes in gonads of infertile mouse models.

## Materials and methods

### Ethics statement

All animal experiments were approved by the local animal experiments committee DEC Consult and animals were maintained under supervision of the Animal Welfare Officer.

### Generation of SYCPC and CSYCP mice

In order to study the dynamics of the SC, we have generated transgenic mice expressing N- or C- terminal fusions of SYCP3 with the mCherry protein. To this end, we first cloned the coding region of the mouse *Sycp3* gene into the mCherry C1 or N1 vector. The mouse *Sycp3* coding region was amplified using forward primer 5′CCGCTCGAGTGCTTCGAGGGTGTG3′ and reversed primer 5′CGCGGATCCAGACTCATCAGAATAACATG 3′ to generate CSYCP and forward primer CCGCTCGAGTCAGATGCTTCGAGGGTG and reversed primer 5′CGCGGATCCCAGAATAACATGGATTGAAGAG 3′ to generate SYCPC. Both fragments were cut with XhoI and BamH1 and cloned into the multiple cloning site of the C1 and N1 vector, respectively. Subsequently, we amplified a 295 bp fragment of the *Smc1β*-promoter previously described to drive expression from leptotene onwards (Adelfalk et al., 2009) using PCR primers SMC1bFor: 5′ CCGCTATTAATCACGGCAAGAAAAGCCC 3′ and SMC1bRev: 5′ CTAGCTAGCGACCGGTGCCTCAGCC 3′ followed by digestion of the fragment with AseI and NheI and cloning into the two vectors containing the SYCPC and CSYCP constructs by replacing the CMV promoter in these plasmids. Finally, the constructs were digested with EciI, purified and injected in the pronuclei of FVB zygotes using standard methods. Transgenic mice were tested for expression of the transgenes in the testes, and the SYCP3 open reading frame was sequenced to exclude point mutations. Mice carrying the transgenes could be bred to homozygosity without affecting overall health. Animals were genotyped using standard DNA isolation (DNA isolated from tail or toe tips) and PCR procedures using the following primer sets: forward primer 5′CACCATCGTGGAACAGTACG 3′ and reverse primer 5′GGGAGGTGTGGGAGGTTTT3′ for CSYCP and forward primer 5′GCAAGGGCGAGGAGGATAAC 3′ and reverse primer 5′ TTGCCGATTTCGGCCTATTG 3′ for SYCPC.

### Mice

*Sycp3* knockout mice were described previously (Yuan et al. 2000) and crossed with SYCPC or CSYCP mice. Testes were isolated from immature and adult male mice. To obtain ovaries containing oocytes at different stages of meiotic prophase, *Sycp3*^*+/−*^ (or occasionally *Sycp3*^*−/−*^in case of females) with or without the CSYCP or SYCPC transgene were mated and ovaries were isolated from embryos at E.15.5, 16.5, or E18.5. (the day at which a plug was observed was considered as day 0.5 of embryonic development, E0.5). Meiotic progression in females differs in terms of timing and stage appearance compared to the male. The more or less synchronous development of oocytes in the embryonic ovary allowed us to predict in which stage the majority of the cells would be depending on the embryonic day of development. In this way, the majority of the oocytes will be at leptotene or zygotene at E15.5 and E16.5 and at pachytene or diplotene at E18.5 (Ashley 2004; Dietrich and Mulder 1983).

### Western blotting

Testes were isolated from SYCPC and CSYCP mice of different ages (SYCPC 11, 16, and 19 days old, CSYCP 12, 15, and 22 days old). Total testis protein isolation and Western blotting of 12% SDS-PAGE gels were performed as described previously (Mulugeta Achame et al. 2010). Rabbit polyclonal anti-SYCP3 (Lammers et al. 1994) was diluted 1:5000. Peroxidase-labeled secondary antibody (Sigma) was used, and antigen-antibody complexes were detected using a chemoluminescence kit (Du Pont/NEN, Bad Homburg, Germany) according to the instructions provided by the manufacturer.

### Immunocytochemistry

Spread nuclei of spermatocytes and oocytes were prepared from isolated gonads as described by Peters et al. (1997) and stored at − 80C. Thawed slides were washed in PBS (3 × 10 min), and non-specific sites were blocked with 0.5% *w*/*v* BSA and 0.5% *w*/*v* milk powder in PBS. Primary antibodies were diluted in 10% *w*/*v* BSA in PBS, and incubations were performed overnight at room temperature in a humid chamber. Subsequently, slides were washed (3 × 10 min) in PBS, blocked in 10% *v*/*v* normal goat serum (Sigma) in blocking buffer (supernatant of 5% *w*/*v* milk powder in PBS centrifuged at 14,000 rpm for 10 min), and incubated with secondary antibodies in 10% normal goat serum in blocking buffer at room temperature for 2 h. Finally, slides were washed (3 × 10 min) in PBS and embedded in Prolong Gold with DAPI (Invitrogen). For stainings involving two primary antibodies generated in the same species, the immunostaining was performed sequentially. After adding one of the two primary antibodies, and its detection on the next day with a secondary antibody, the other primary antibody was added and detected the day thereafter with a secondary antibody of a different color. Note that the protein detected by the antibody added on the first day would be visible in the colors of each of the two secondary antibodies, while the protein detected by the primary antibody that was added on the second day would be visible only in the color of the last secondary antibody.

Primary antibodies: mouse monoclonal anti-SYCP3 (ABCAM:ab97672) at 1:200, rabbit polyclonal anti-SYCP3 (Lammers et al. 1994) at 1:10000, guinea pig polyclonal anti-SYCP2 (Yang et al. 2006) at 1:100, rabbit polyclonal anti-SYCP2 (Offenberg et al. 1998) at 1:400, rabbit polyclonal anti-RAD51 (Essers et al. 2002) at 1:500, rabbit polyclonal anti-REC8 (N-terminus, affinity purified) at 1:50 (Eijpe et al. 2003), rabbit polyclonal anti-SYCP1 (Meuwissen et al. 1992) at 1:5000, mouse monoclonal anti-MLH1 (cat. 551,091, BD Pharmigen) at 1:25. For secondary antibodies, we used a goat anti-rabbit alexa 488 IgG, goat anti-rabbit alexa 633 IgG, goat anti-mouse alexa 488, and goat anti-mouse alexa 546 IgG, all at 1:500 dilution.

Fluorescent images were obtained using a fluorescence microscope (Axioplan 2; Carl Zeiss) equipped with a digital camera (Coolsnap-Pro; Photometrics). Confocal images were taken using a Zeiss LSM700 confocal, equipped with a digital camera (Axiocam MRm Rev.3 1388X1040 monochrome camera for epi-fluorescence). Foci were counted using ImageJ software (FIJI); we counted foci using the find maxima function and set the noise tolerance manually.

### Staging of spermatocytes and oocytes

Staging of wild-type spermatocytes and oocytes was based on the pattern of the SYCP3 or REC8 (*Sycp3*^*−/−*^ spermatocytes and oocytes). In addition, RAD51 was used in order to distinguish between zygotene (250–100 foci, as the number of foci decreases as synapsis progresses) and diplotene oocytes (< 10 foci (Moens et al. 1997). Alternatively, the presence of MLH1 foci was used as marker of pachytene and diplotene oocytes.

### Isolation of testis tubules and embryonic ovaries for culture

Adult mouse testes from transgenic mice carrying the CSYCP or SYCPC transgene were isolated and dissected following a previously described protocol (van der Laan et al. 2004) with some modifications. Decapsulated testes were immersed in 20 ml Dulbecco’s phosphate-buffered saline (Invitrogen, Carlsbad, CA, USA) containing 1.1 mM Ca^2+^, 0.52 mM Mg2^+^, 6 mM DL-lactic acid, and 5.6 mM glucose (PBS^+^), in the presence of collagenase (1 μg/μl, type 1 Worthington) and hyaluronidase (0.5 μg/μl, H3884 Sigma) in a 50-ml falcon tube. The testes were then incubated in a water bath at 33 °C and shaken at 90 cycles/min, amplitude of 20 mm, for 5 min. The incubation was stopped when the enzymatic digestion had resulted in dissociation of the interstitial tissue and disengagement of testis tubules. Tubules were separated from interstitial cells by washing in PBS^+^ twice. Next, tubules were placed in a 30-mm BSA-coated Petri dish, where the tubules were separated from each other using dissection tweezers (Nr. 5). After cutting small fragments, these were carefully transferred to a 15-mm BSA-coated Petri dish. After collecting a pool of fragments, we used dissection tweezers (Nr. 5) to place 4–5 of these fragments into a 50-μl drop containing 1:4 *v*/*v* (RPMI + 10% KRS culture medium)/Cultrex® Basement Membrane Extract (BME), type 2; Trevigen pipetted onto a 24-mm laminin-coated cover slip in each live cell chamber. The chambers with the tubules were centrifuged 5 min at 500 rpm and 4 °C. Next, the chambers were incubated for 30 min at 33 °C to allow the BME to jellify. Finally, 2 ml of RPMI + 10% KRS culture medium was added. For embryonic ovary culture, pregnant females were killed at different time points after timed matings. Embryos were collected and ovaries were isolated and placed temporarily in PBS^+^ (without DL-lactic acid). Once all the ovaries had been collected, they were transferred to the live cell chamber, prepared as described for the tubules. The rest of the protocol was also identical, with the exception of the medium (αMEM, Gibco) and the temperature (37 °C instead of 33).

#### Confocal and time-lapse microscopy

For the time-lapse imaging, we used a Leica SP5 confocal microscope. To specifically follow desynapsis in diplotene spermatocytes, we used a Zeiss LSM880 microscope with airy-scan detector. Chamber and objective (40 × 1.25 NA, oil immersion) were kept at 33 °C for seminiferous tubules and 37 °C for ovaries. The chambers with the tubules or ovaries were also maintained at 5% CO_2_. Red fluorescent images were obtained after excitation with a 594-nm laser and detection through a 600–680 band pass filter.

For the FRAP analysis, we bleached a single strip of 16 pixels high (pixel size 0.11 μm XY) that spanned the middle part of the nucleus, for five iterations at high laser intensity (100% of the 594 and 561 nm laser). The recovery of fluorescence in the strips was monitored during 1 min at intervals of 0.020 s at 6–12% of the laser intensity applied for bleaching (12% for the mice carrying the SYCPC transgene and 6% for the mouse carrying the CSYCP transgene, due to its higher signal intensity). Samples of two mice, one carrying CSYCP and the other carrying SYCPC, were processed together and analyzed the same day. Another set of two mice carrying each variant of the transgene was used to confirm the results.

For the overnight time-lapse experiments, we selected 5–10 different positions along each tubule or ovary, and for each position, a stack of approximately 30 slices of 1 μm was made. The lapse between images was 10 min. For the short videos time-lapse experiments, total time and lapse varied between experiments.

### Analysis of the rotation, chromosome movements, and axis separation

Time-lapse videos of one plane per time point and a time lapse of 1.5–2 s were recorded for this analysis. For the rotation in X-Y analysis, single oocytes or spermatocytes were selected. The rotation angle of the time point *t* + 1 with respect to *t* was calculated. For this, the nucleus at *t* + 1 was translated (X,Y) and rotated until the correlation between *t* and *t* + 1 was the highest. From the rotation angle, we calculated the rotation speeds (degrees/s).

To measure the speed of the XY chromosome in pachytene spermatocytes, we measured the distance from the center of the XY at time *t* + 1 to the center at time *t* and divided this value by the time in seconds.

To measure the speed of desynapsis, the length of the remaining synapsed (bivalent b, d) or desynapsed (bivalent a,c, e) fragment was measured at each analyzed time point, in one single XY plane. The length difference between the two time points was measured and used to calculate the speed. Speeds were determined for each 30-s interval during the time period for which the bivalents could be traced.

## Electronic supplementary material

Fig. S1 Generation of transgenic mice expressing N- and C-terminal fusions of SYCP3 to mCherry. (a) Schematic drawing of SYCPC and CSYCP constructs. See Materials and Methods for details. (b) Western blot of total testis protein extracts isolated from mice of different age, stained with anti-SYCP3 antibody. Testis extracts were prepared from SYCPC mice of 11, 16 and 19 days-old, and of CSYCP mice of 12, 15 and 22 days-old. Two bands of around 30 K, representing the endogenous SYCP3 protein are detected at all ages. Expression of both fusion proteins (around 60 K) is initiated later, and the overall expression level is lower than that of endogenous SYCP3 (GIF 51 kb)

High resolution image (TIFF 9136 kb)

Fig. S2 SYCPC and CSYCP expression does not interfere with meiotic progression and fertility in males and females. (a) Box plots of litter sizes from backcrosses of control mice (*Ube2b*^*+/−*^ mice (Roest et al. 1996) (9.1 ± 2.4, SD; *n* = 18 breedings)), and SCYCP (10.1 ± 2.0, SD; *n* = 16 breedings) and CSYCP (8.9 ± 2.7, SD; *n* = 26 breedings) mice to FVB mice. All breedings took place in the same period in our animal facility (period 2009–2012). No significant differences between genotypes were observed (Mann-Whitney U test). Median values are indicated by the horizontal blue lines within each box, mean values are shown in red. The upper and lower whiskers indicate the upper and lower quartiles of the values, respectively. Outliers are shown as green dots. (b) Box plots of MLH1 foci numbers in late pachytene/early diplotene nuclei of control (*Sycp3*^*+/−*^ (23.6 ± 1.7,SD; *n* = 10 nuclei))*,* two *Sycp3*^*+/−*^ SYCPC (22.8 ± 1.3,SD and 23.9 ± 1.6,SD; *n* = 12 and 11, respectively),and two *Sycp3*^*+/−*^ CSYCP (25 ± 2.8,SD and 26.4 ± 2.5, SD; *n* = 11 and 13, respectively) male mice. Only a single *Sycp3*^*+/−*^ CSYCP mouse displayed a slight increase in MLH1 foci number compared to the Sycp3^*+/−*^ and Sycp3^*+/−*^ SYCPC mice (*p* = 0.009, Mann-Whitney U test). (c) Percentage of late pachytene/diplotene nuclei displaying one or more H2AX domains in addition to the XY body in one control (*Sycp3*^*+/−*^), two *Sycp3*^*+/−*^ SYCPC and two *Sycp3*^*+/−*^ CSYCP mice. We observed no statistically significant effect of the transgenes on the persistence of DNA damage (Mann Whitney U test, *n* = 100 nuclei for each mouse). (GIF 34 kb)

High resolution image (TIFF 7230 kb)

Fig. S3 SYCP2 is expressed as axial element component in *Sycp3*^*−/−*^ spermatocyte nuclei. Immunostaining of SYCP2 (green) on spermatocyte nuclei using two different antibodies: rabbit anti-SYCP2 (SYCP2 rb) and guinea pig anti-SYCP2 (SYCP2 gp) . (a) Wild type nuclei at indicated prophase stages (b) Zygotene-like nuclei of *Sycp3*^−/−^, *Sycp3*^−/−^ SYCPC, and *Sycp3*^−/−^ CSYCP mice. Scale bar 10 μm (GIF 81 kb)

High resolution image (TIFF 22310 kb)

Fig. S4 Localisation pattern of tagged SYCP3 in E16.5 oocytes. (a) *Sycp3*^−/−^ SYCPC (b) *Sycp3*^−/−^ CSYCP. Immunostaining of REC8 (green) and SYCP3 (red). Scale bar 10 μm (GIF 124 kb)

High resolution image (TIFF 23559 kb)

Fig. S5 Immunostaining of REC8 (green) and RAD51 (white) on *Sycp3*^*−/−*^ (a), *Sycp3*^−/−^ SYCPC (b) and *Sycp3*^−/−^ CSYCP (c) oocyte nuclei at E18.5. Means of RAD51 foci number ± SD in diplotene-like stage are displayed in the images (*N* = 6 nuclei for *Sycp3*^*+/−*^ SYCPC and *Sycp3*^*+/−*^ CSYCP; *n* = 8 nuclei for *Sycp3*^*−/−*^ and *Sycp3*^*−/−*^ SYCPC; n = 11 nuclei for *Sycp3*^*−/−*^ CSYCP). Scale bar 10 μm (GIF 58 kb)

High resolution image (TIFF 13492 kb)

Fig. S6 Bouquet progression of the N3 cyst. Each image represents the single plane of each nucleus that showed the clearest telomere organization. At some time points this required displaying each nucleus from a separate plane; these were then merged in one image for a better visualization (T1-T7, T9, T10, T13, T21, T25, T34, T37, T38 and T44). Scale bar 5 μm. Nuclei are labeled A and B. If a bouquet configuration is observed, these letters are red. (T0- T14) time previous to the formation of the bouquet. A one-time point clustering can be observed at T3 in nucleus B. (T15-T27) Bouquet in A. (T15-T19 and T23-T25). Bouquet in B. One time point clustering for both of the nuclei at T45 and for A at T49. Reclustering of nucleus A at T51 till T59, for nucleus B at T54 till T55. Some 1-time point dissolutions of nucleus A at T57 and T59. (GIF 293 kb)

High resolution image (TIFF 25494 kb)

Video 1 Two leptotene oocytes at E16.5 sharing the same cyst. 1 plane, 2 s interval. Total recording time 2 min (AVI 7151 kb)

Video 2 Two late zygotenes oocytes at E16.5 sharing the same cyst. 1 plane, 2 s interval. Total recording time 2 min (AVI 8306 kb)

Video 3 Three zygotenes oocytes at E16.5 sharing the same cyst. Stack, 5 s interval. Total recording 2 min (AVI 1604 kb)

Video 4 Two zygotenes oocytes at E16.5 sharing the same cyst. At *t* = 6 s, a bouquet configuration can be clearly observed. 1 plane, 3 s interval. Total recording 12 s (AVI 44 kb)

Video 5 Desynapsis progression in a *Sycp3*^*+/−*^ CSYCP diplotene spermatocyte ((a) in Fig. 7). Arrow heads indicate desynapsed sites, the position indicated with a green arrow was followed. No desynapsis progression was observed from the position indicated with a red arrow. 1 plane, 30 s interval, total recording 4 min 30 s (AVI 621 kb)

Video 6 Desynapsis progression in a *Sycp3*^*+/−*^ CSYCP diplotene spermatocyte ((b) in Fig. 7). The green arrow heads indicate the position where desynapsis/resynapsis take place. 1 plane, 30 s interval, total recording 4 min 30 s (AVI 347 kb)

Video 7 Desynapsis progression in a *Sycp3*^*+/−*^ CSYCP diplotene spermatocyte ((c) in Fig. 7). Green arrow head indicates the last synapsed part of the bivalent where the desynapsis occurs. 1 plane, 30 s interval, total recording 4 min 30 s (AVI 588 kb)

Video 8 Desynapsis progression in a *Sycp3*^*+/−*^ CSYCP diplotene spermatocyte ((d) in Fig. 7). The green arrow heads indicate the position where desynapsis occurs. 1 plane, 30 s interval, total recording 4 min 30 s. (AVI 249 kb)

Video 9 Desynapsis progression in a *Sycp3*^*+/−*^ CSYCP diplotene spermatocyte ((e) in Fig. 7). The green arrow heads indicate the position where desynapsis/resynapsis take place. 1 plane, 30 s interval, total recording 4 min 30 s (AVI 676 kb)
