# Supplementary figures and images for: Live cell analyses of synaptonemal complex dynamics and chromosome movements in cultured mouse testis tubules and embryonic ovaries
Source: Chromosoma. 2018 Mar 26;127(3):341–59. doi: 10.1007/s00412-018-0668-7 (PMC6096571; doi:10.1007/s00412-018-0668-7)

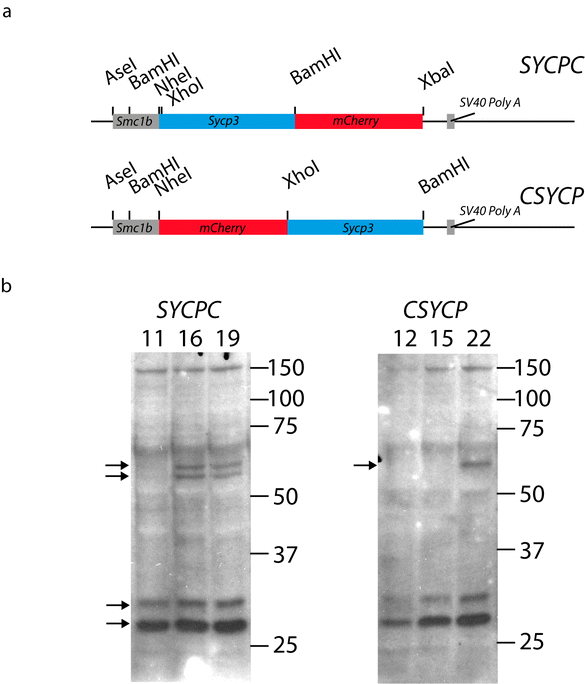

Supplement: Supplementary file 1 — Generation of transgenic mice expressing N- and C-terminal fusions of SYCP3 to mCherry. (a) Schematic drawing of SYCPC and CSYCP constructs. See Materials and Methods for details. (b) Western blot of total testis protein extracts isolated from mice of different age, stained with anti-SYCP3 antibody. Testis extracts were prepared from SYCPC mice of 11, 16 and 19 days-old, and of CSYCP mice of 12, 15 and 22 days-old. Two bands of around 30 K, representing the endogenous SYCP3 protein are detected at all ages. Expression of both fusion proteins (around 60 K) is initiated later, and the overall expression level is lower than that of endogenous SYCP3 (GIF 51 kb) [file 412_2018_668_Fig9_ESM.gif]

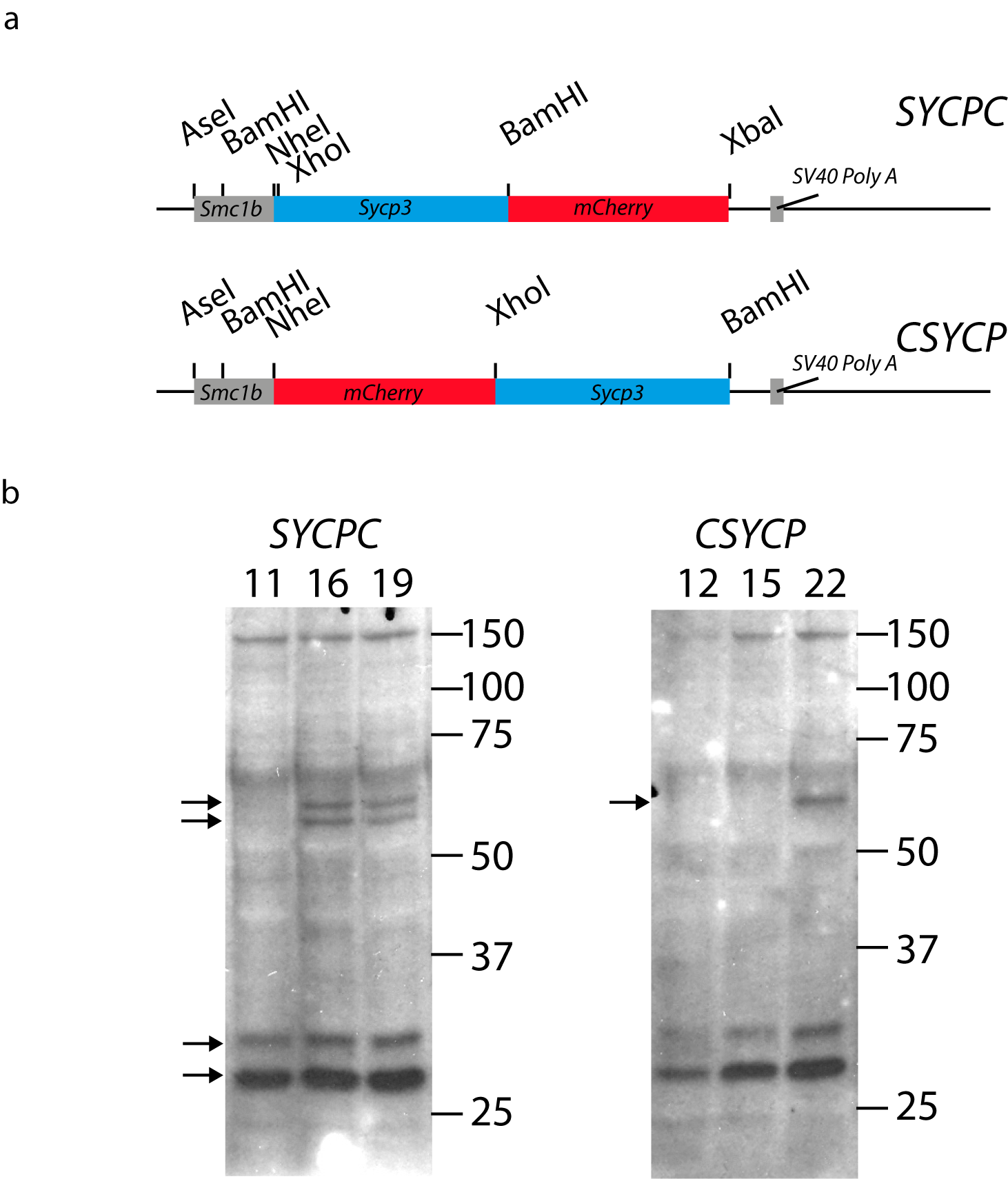

Supplement: Supplementary file 2 — High resolution image (TIFF 9136 kb) [file 412_2018_668_MOESM1_ESM.tif]

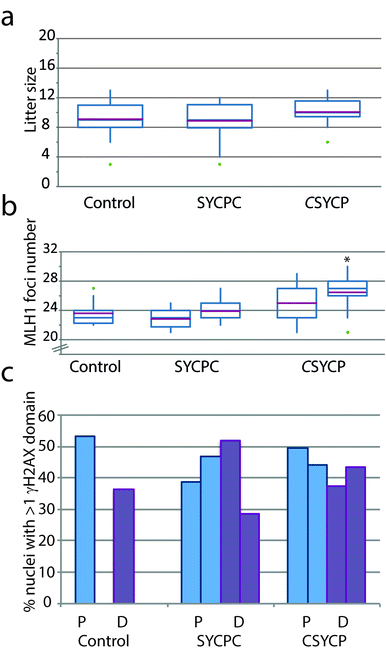

Supplement: Supplementary file 3 — SYCPC and CSYCP expression does not interfere with meiotic progression and fertility in males and females. (a) Box plots of litter sizes from backcrosses of control mice (Ube2b+/− mice (Roest et al. 1996) (9.1 ± 2.4, SD; n = 18 breedings)), and SCYCP (10.1 ± 2.0, SD; n = 16 breedings) and CSYCP (8.9 ± 2.7, SD; n = 26 breedings) mice to FVB mice. All breedings took place in the same period in our animal facility (period 2009–2012). No significant differences between genotypes were observed (Mann-Whitney U test). Median values are indicated by the horizontal blue lines within each box, mean values are shown in red. The upper and lower whiskers indicate the upper and lower quartiles of the values, respectively. Outliers are shown as green dots. (b) Box plots of MLH1 foci numbers in late pachytene/early diplotene nuclei of control (Sycp3+/− (23.6 ± 1.7,SD; n = 10 nuclei)), two Sycp3+/− SYCPC (22.8 ± 1.3,SD and 23.9 ± 1.6,SD; n = 12 and 11, respectively),and two Sycp3+/− CSYCP (25 ± 2.8,SD and 26.4 ± 2.5, SD; n = 11 and 13, respectively) male mice. Only a single Sycp3+/− CSYCP mouse displayed a slight increase in MLH1 foci number compared to the Sycp3+/− and Sycp3+/− SYCPC mice (p = 0.009, Mann-Whitney U test). (c) Percentage of late pachytene/diplotene nuclei displaying one or more H2AX domains in addition to the XY body in one control (Sycp3+/−), two Sycp3+/− SYCPC and two Sycp3+/− CSYCP mice. We observed no statistically significant effect of the transgenes on the persistence of DNA damage (Mann Whitney U test, n = 100 nuclei for each mouse). (GIF 34 kb) [file 412_2018_668_Fig10_ESM.gif]

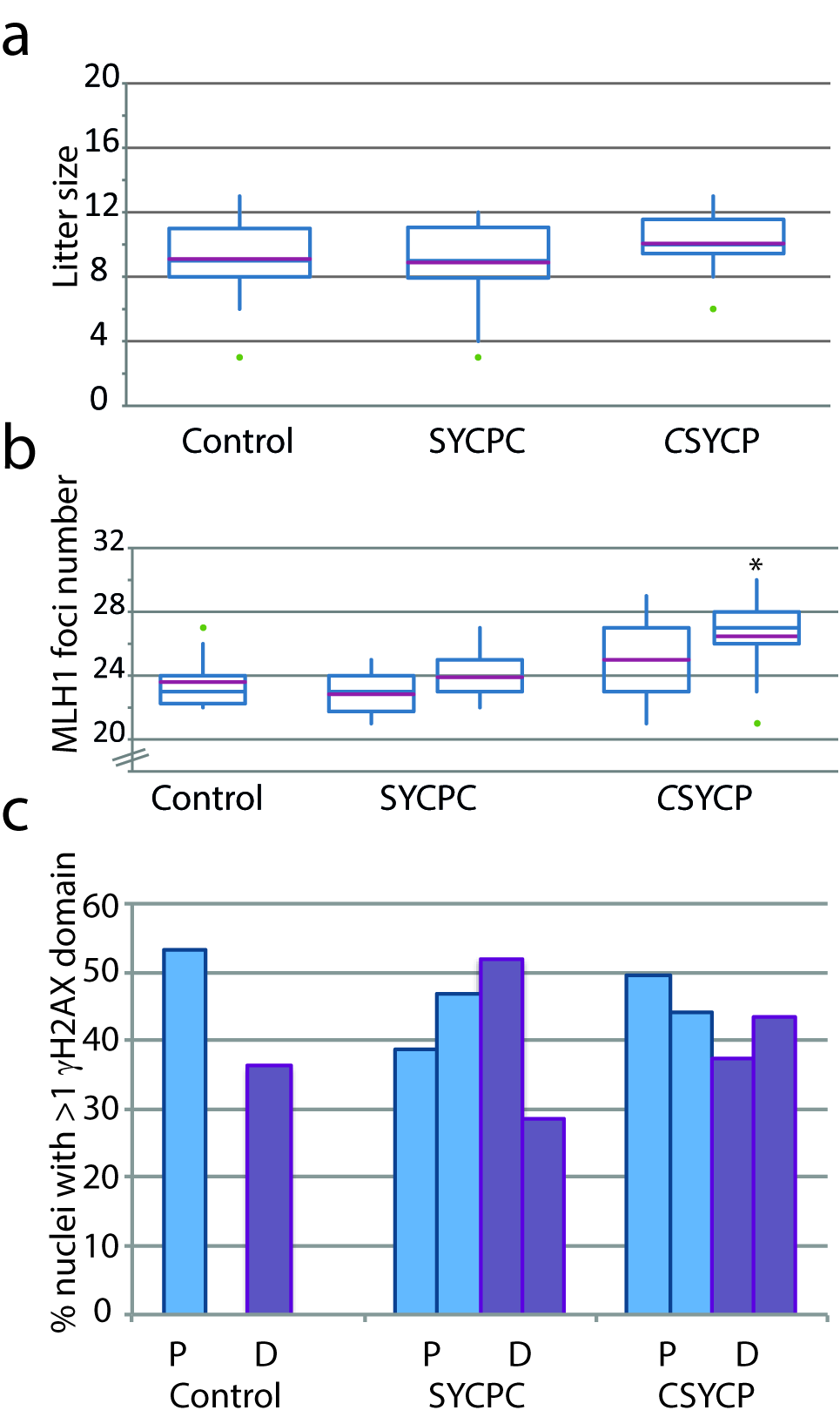

Supplement: Supplementary file 4 — High resolution image (TIFF 7230 kb) [file 412_2018_668_MOESM2_ESM.tif]

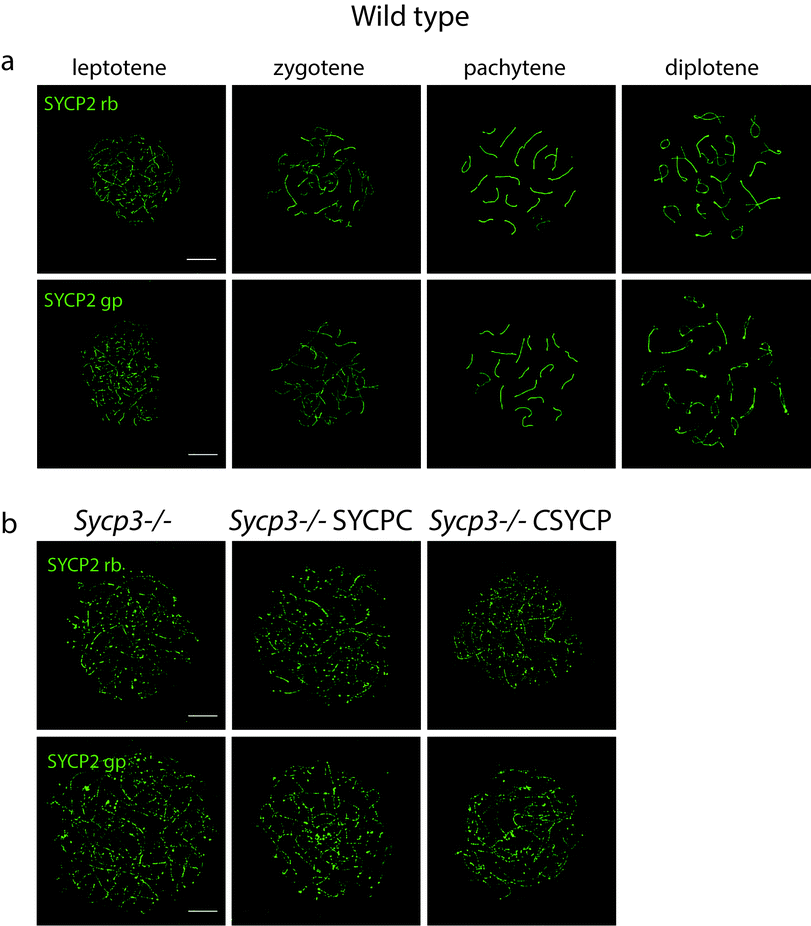

Supplement: Supplementary file 5 — SYCP2 is expressed as axial element component in Sycp3−/− spermatocyte nuclei. Immunostaining of SYCP2 (green) on spermatocyte nuclei using two different antibodies: rabbit anti-SYCP2 (SYCP2 rb) and guinea pig anti-SYCP2 (SYCP2 gp) . (a) Wild type nuclei at indicated prophase stages (b) Zygotene-like nuclei of Sycp3−/−, Sycp3−/− SYCPC, and Sycp3−/− CSYCP mice. Scale bar 10 μm (GIF 81 kb) [file 412_2018_668_Fig11_ESM.gif]

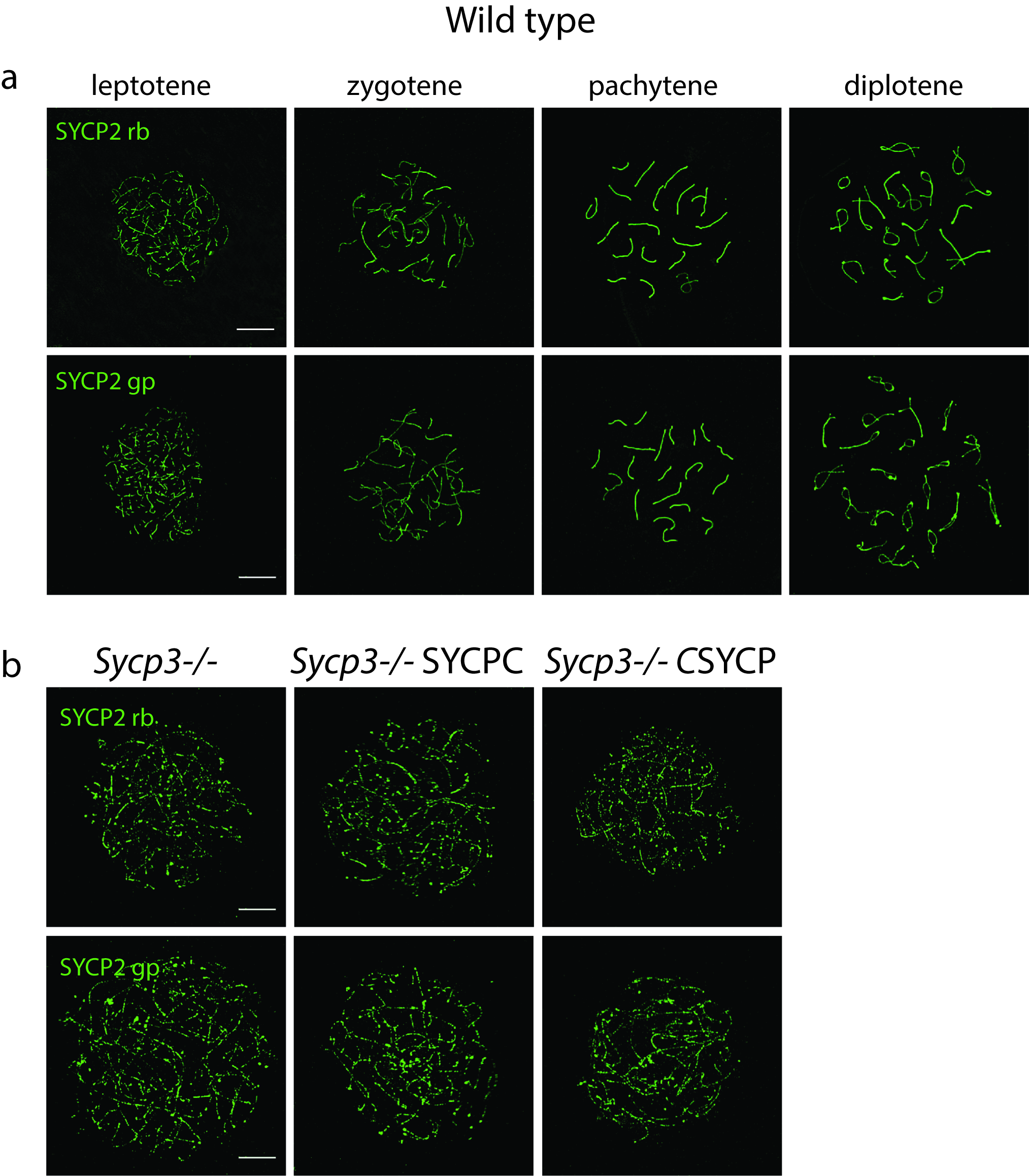

Supplement: Supplementary file 6 — High resolution image (TIFF 22310 kb) [file 412_2018_668_MOESM3_ESM.tif]

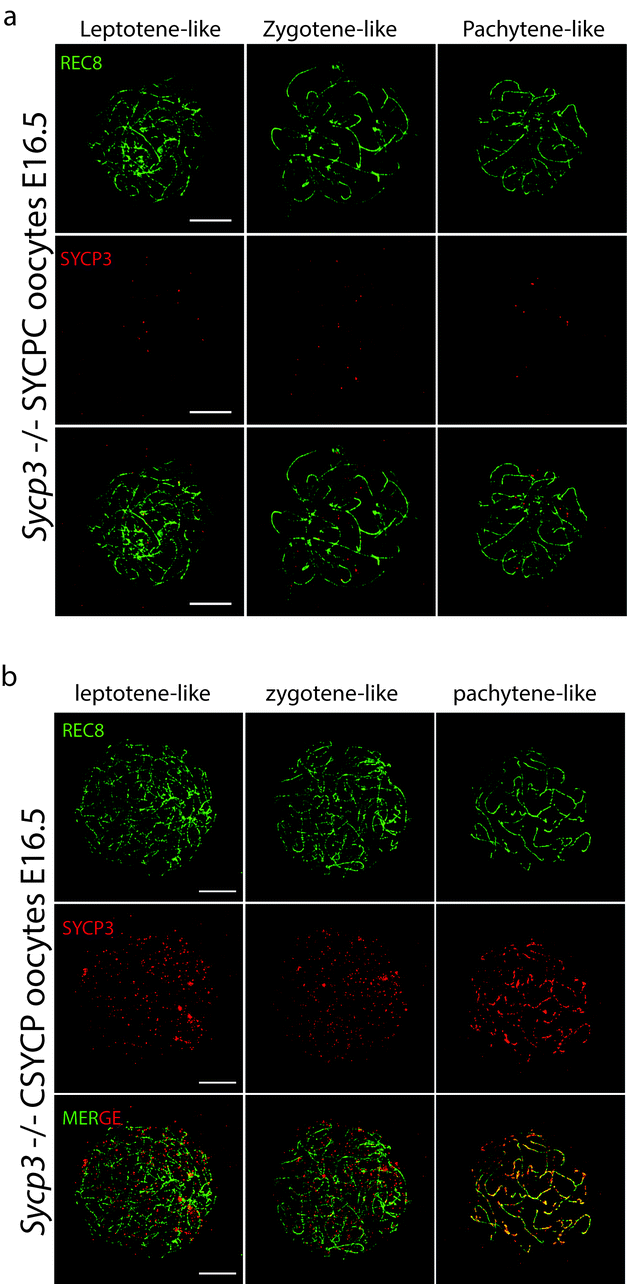

Supplement: Supplementary file 7 — Localisation pattern of tagged SYCP3 in E16.5 oocytes. (a) Sycp3−/− SYCPC (b) Sycp3−/− CSYCP. Immunostaining of REC8 (green) and SYCP3 (red). Scale bar 10 μm (GIF 124 kb) [file 412_2018_668_Fig12_ESM.gif]

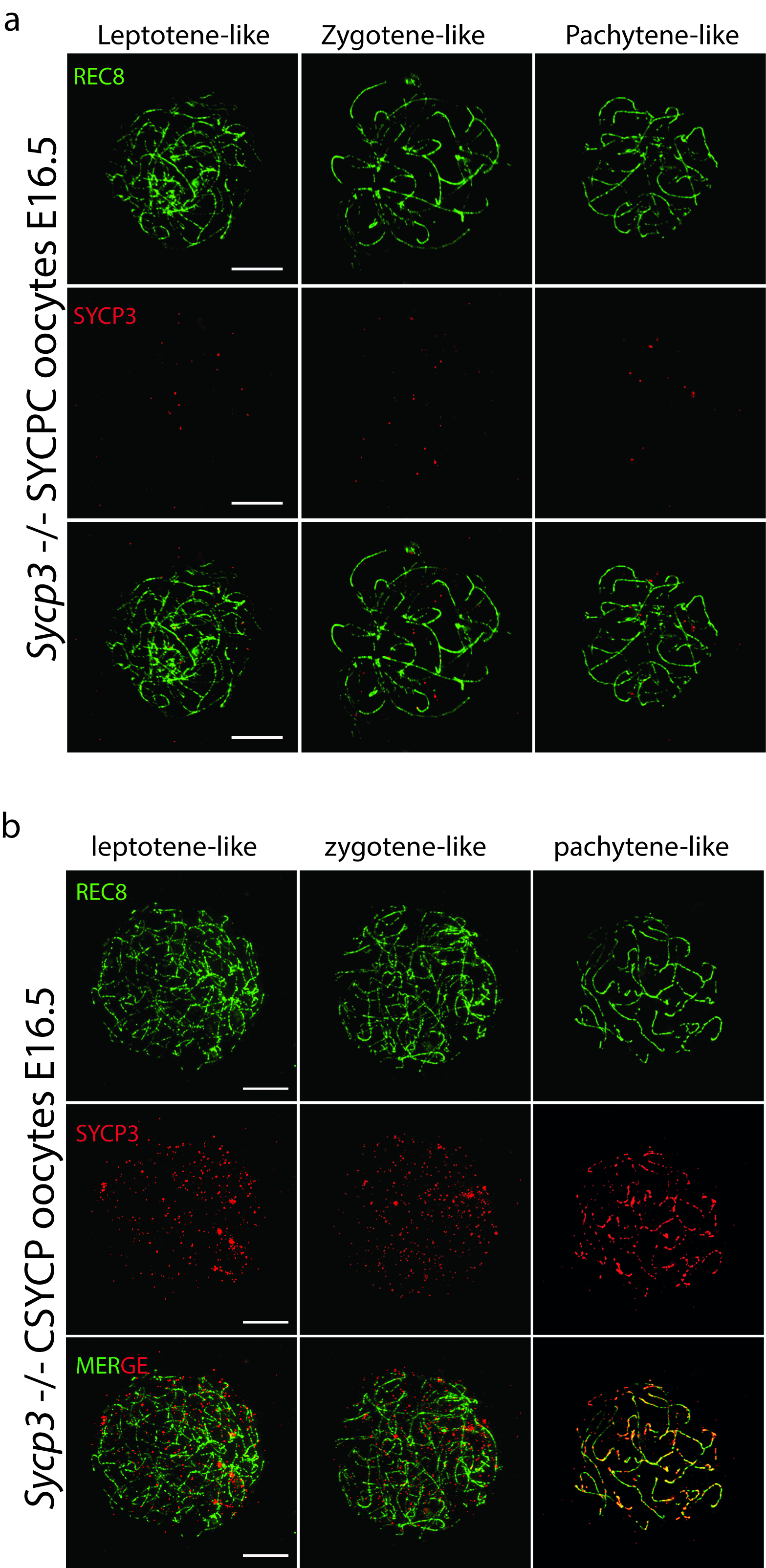

Supplement: Supplementary file 8 — High resolution image (TIFF 23559 kb) [file 412_2018_668_MOESM4_ESM.tif]

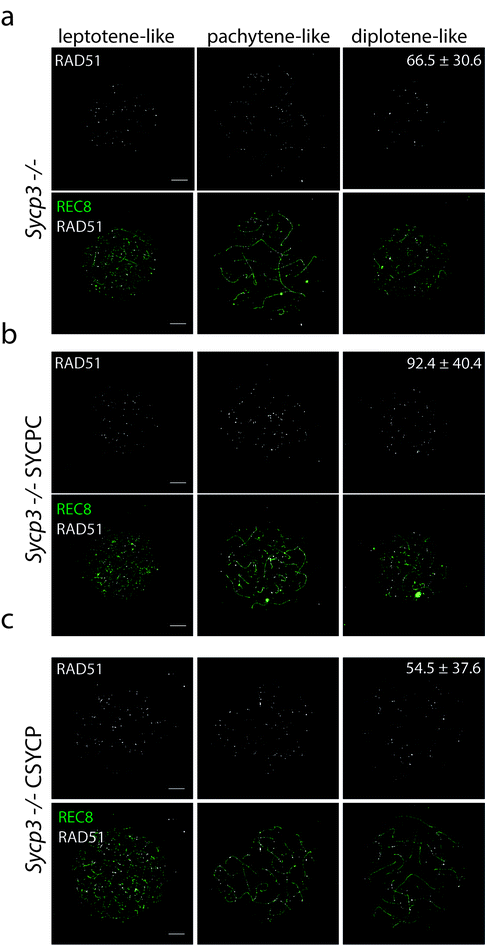

Supplement: Supplementary file 9 — Immunostaining of REC8 (green) and RAD51 (white) on Sycp3−/− (a), Sycp3−/− SYCPC (b) and Sycp3−/− CSYCP (c) oocyte nuclei at E18.5. Means of RAD51 foci number ± SD in diplotene-like stage are displayed in the images (N = 6 nuclei for Sycp3+/− SYCPC and Sycp3+/− CSYCP; n = 8 nuclei for Sycp3−/− and Sycp3−/− SYCPC; n = 11 nuclei for Sycp3−/− CSYCP). Scale bar 10 μm (GIF 58 kb) [file 412_2018_668_Fig13_ESM.gif]

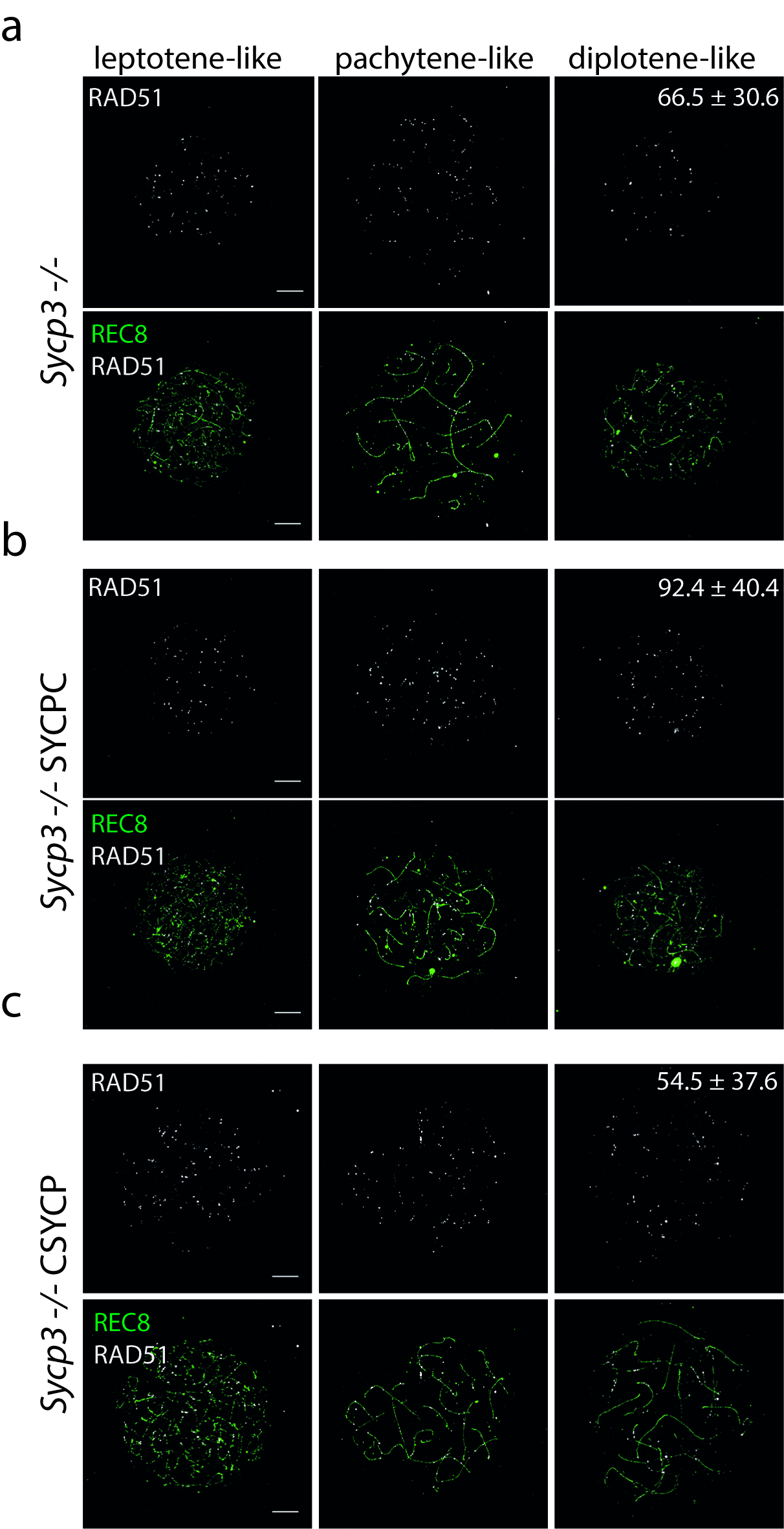

Supplement: Supplementary file 10 — High resolution image (TIFF 13492 kb) [file 412_2018_668_MOESM5_ESM.tif]

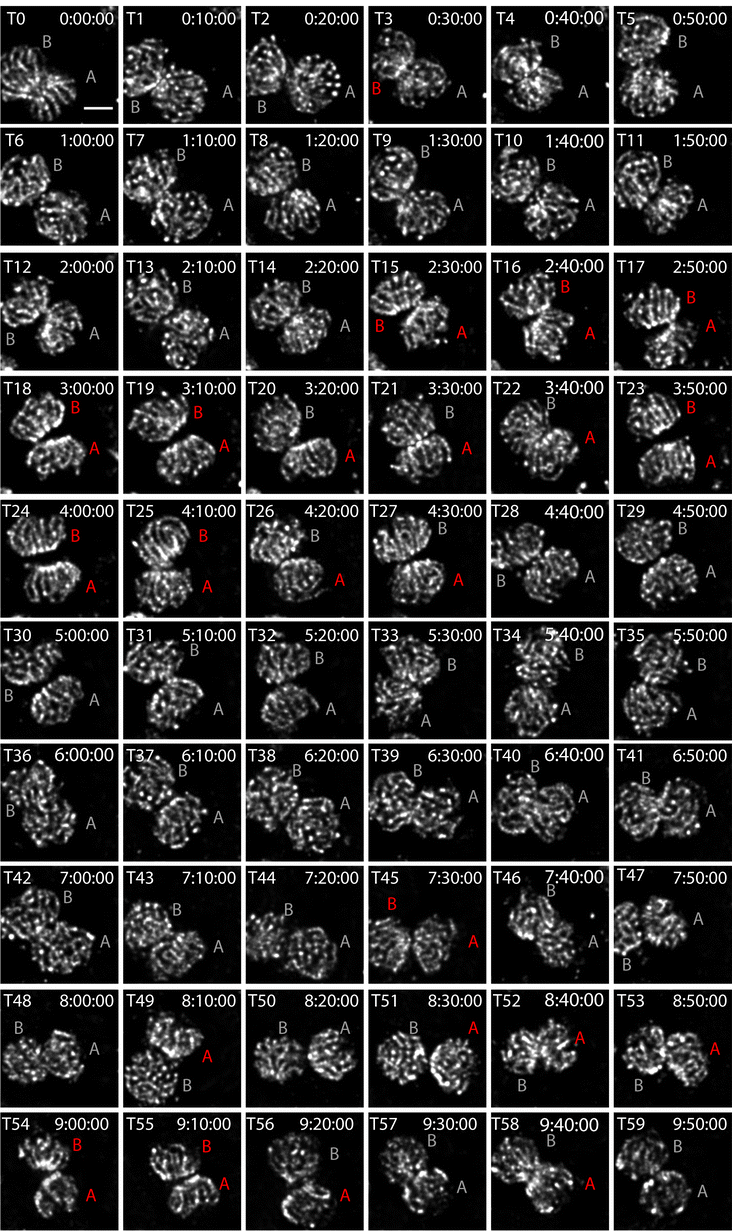

Supplement: Supplementary file 11 — Bouquet progression of the N3 cyst. Each image represents the single plane of each nucleus that showed the clearest telomere organization. At some time points this required displaying each nucleus from a separate plane; these were then merged in one image for a better visualization (T1-T7, T9, T10, T13, T21, T25, T34, T37, T38 and T44). Scale bar 5 μm. Nuclei are labeled A and B. If a bouquet configuration is observed, these letters are red. (T0- T14) time previous to the formation of the bouquet. A one-time point clustering can be observed at T3 in nucleus B. (T15-T27) Bouquet in A. (T15-T19 and T23-T25). Bouquet in B. One time point clustering for both of the nuclei at T45 and for A at T49. Reclustering of nucleus A at T51 till T59, for nucleus B at T54 till T55. Some 1-time point dissolutions of nucleus A at T57 and T59. (GIF 293 kb) [file 412_2018_668_Fig14_ESM.gif]

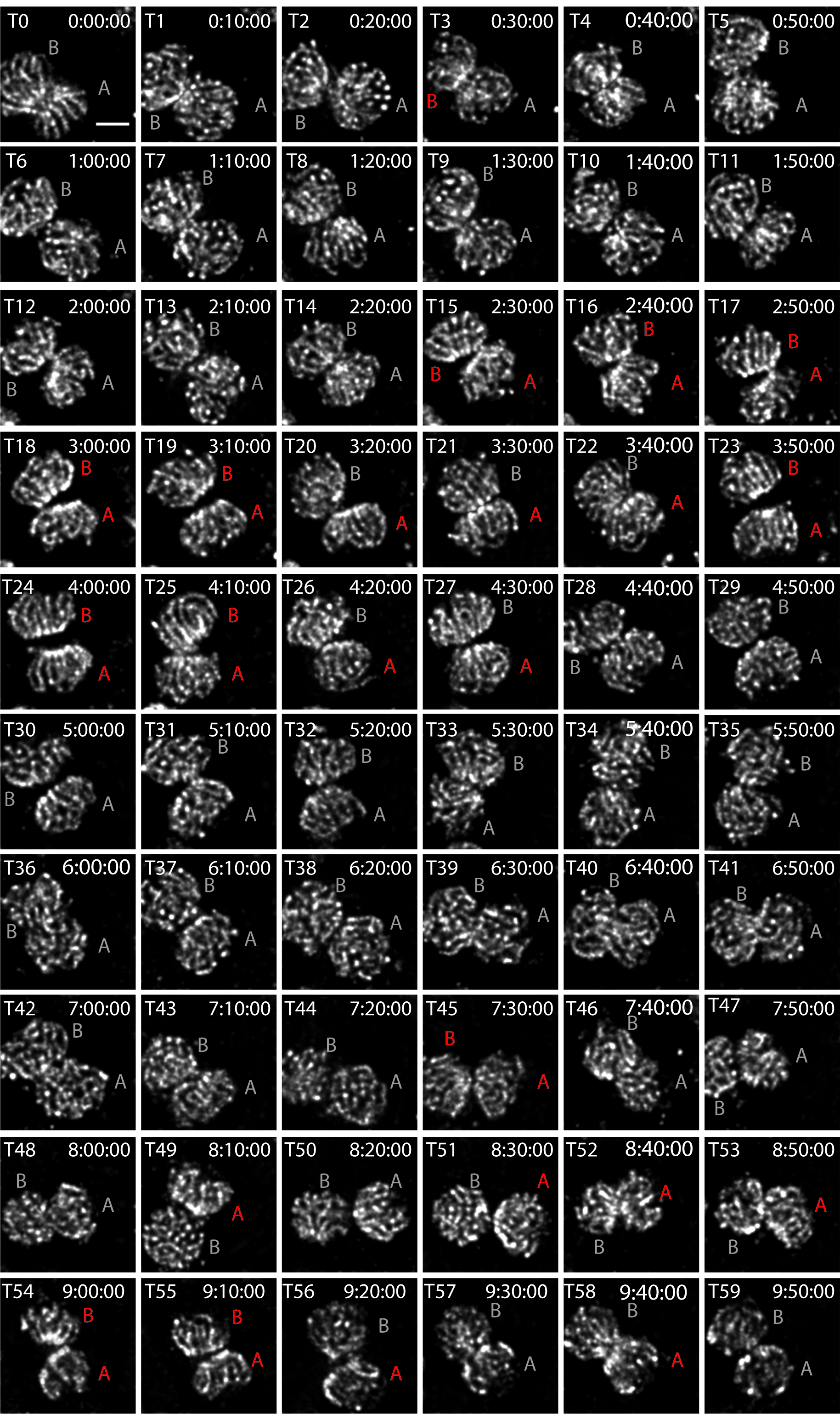

Supplement: Supplementary file 12 — High resolution image (TIFF 25494 kb) [file 412_2018_668_MOESM6_ESM.tif]
